# The global spread of HIV-1 subtype B epidemic

**DOI:** 10.1016/j.meegid.2016.05.041

**Published:** 2016-12

**Authors:** Gkikas Magiorkinis, Konstantinos Angelis, Ioannis Mamais, Aris Katzourakis, Angelos Hatzakis, Jan Albert, Glenn Lawyer, Osamah Hamouda, Daniel Struck, Jurgen Vercauteren, Annemarie Wensing, Ivailo Alexiev, Birgitta Åsjö, Claudia Balotta, Perpétua Gomes, Ricardo J. Camacho, Suzie Coughlan, Algirdas Griskevicius, Zehava Grossman, Anders Horban, Leondios G. Kostrikis, Snjezana J. Lepej, Kirsi Liitsola, Marek Linka, Claus Nielsen, Dan Otelea, Roger Paredes, Mario Poljak, Elizabeth Puchhammer-Stöckl, Jean Claude Schmit, Anders Sönnerborg, Danica Staneková, Maja Stanojevic, Dora C. Stylianou, Charles A.B. Boucher, on behalf of the SPREAD program, Georgios Nikolopoulos, Tetyana Vasylyeva, Samuel R. Friedman, David van de Vijver, Gioacchino Angarano, Marie-Laure Chaix, Andrea de Luca, Klaus Korn, Clive Loveday, Vincent Soriano, Sabine Yerly, Mauricio Zazzi, Anne-Mieke Vandamme, Dimitrios Paraskevis

**Affiliations:** a Department of Zoology, University of Oxford, United Kingdom; b Department of Hygiene, Epidemiology and Medical Statistics, Medical School, National and Kapodistrian University of Athens, Greece; c Department of Microbiology, Tumor and Cell Biology, Karolinska Institutet, Stockholm, Sweden; d Department of Computational Biology, Max Planck Institute for Informatics, Saarbrücken, Germany; e Robert Koch-Institute, Berlin, Germany; f Centre de Recherche Public de la Sante, Luxembourg, Luxembourg; g Clinical and Epidemiological Virology, Rega Institute for Medical Research, Department of Microbiology and Immunology, KU Leuven, Leuven, Belgium; h Department of Virology, University Medical Center, Utrecht, The Netherlands; i National Center of Infectious and Parasitic Diseases, Sofia, Bulgaria; j University of Bergen, Bergen, Norway; k University of Milan, Milan, Italy; l Molecular Biology Lab, LMCBM, SPC, HEM, Centro Hospitalar de Lisboa Ocidental, Lisbon, Portugal; m University College Dublin, Dublin, Ireland; n Lithuanian AIDS Center, Vilnius, Lithuania; o Tel Aviv University, Tel Aviv, Israel; p Hospital of Infectious Diseases, Warsaw, Poland; q University of Cyprus, Nicosia, Cyprus; r Department of Molecular Diagnostics and Flow Cytometry, University Hospital for Infectious Diseases “Dr. F. Mihaljevic”, Zagreb, Croatia; s National Institute of Health and Welfare, Helsinki, Finland; t National Reference Laboratory of AIDS, National Institute of Health, Prague, Czech Republic; u Statens Serum Institute, Copenhagen, Denmark; v National Institute for Infectious Diseases “Prof. Dr. Matei Bals”, Bucharest, Romania; w IrsiCaixa Foundation, Badalona, Spain; x Slovenian HIV/AIDS Reference Centre, University of Ljubljana, Faculty of Medicine, Ljubljana, Slovenia; y University of Vienna, Vienna, Austria; z Department of Clinical Microbiology, Karolinska University Hospital, Stockholm, Sweden; aa Divisions of Infectious Diseases and Clinical Virology, Karolinska Institute, Stockholm, Sweden; ab Slovak Medical University, Bratislava, Slovakia; ac University of Belgrade Faculty of Medicine, Belgrade, Serbia; ad Erasmus MC, University Medical Center, Rotterdam, The Netherlands; ae Institute of Infectious Diseases Research, National Development and Research Institutes, Inc., New York, USA; af Eijkman Winkler Institute, Department of Virology, University Medical Center Utrecht, Utrecht, The Netherlands; ag University of Foggia, Foggia, Italy; ah Laboratoire de Virology, Hôpital Necker Paris, France; ai Institute of Clinical Infectious Diseases, Catholic university, Rome, Italy; aj University of Erlangen, Erlangen, Germany; ak International Clinical Virology Centre, Buckinghamshire, England, United Kingdom; al Hospital Carlos III, Madrid, Spain; am Geneva University Hospital, Geneva, Switzerland; an University of Siena, Siena, Italy

**Keywords:** HIV-1, Subtype B, Phylogeography, Migration pattern, Migration

## Abstract

Human immunodeficiency virus type 1 (HIV-1) was discovered in the early 1980s when the virus had already established a pandemic. For at least three decades the epidemic in the Western World has been dominated by subtype B infections, as part of a sub-epidemic that traveled from Africa through Haiti to United States. However, the pattern of the subsequent spread still remains poorly understood. Here we analyze a large dataset of globally representative HIV-1 subtype B strains to map their spread around the world over the last 50 years and describe significant spread patterns. We show that subtype B travelled from North America to Western Europe in different occasions, while Central/Eastern Europe remained isolated for the most part of the early epidemic. Looking with more detail in European countries we see that the United Kingdom, France and Switzerland exchanged viral isolates with non-European countries than with European ones. The observed pattern is likely to mirror geopolitical landmarks in the post-World War II era, namely the rise and the fall of the Iron Curtain and the European colonialism. In conclusion, HIV-1 spread through specific migration routes which are consistent with geopolitical factors that affected human activities during the last 50 years, such as migration, tourism and trade. Our findings support the argument that epidemic control policies should be global and incorporate political and socioeconomic factors*.*

## Highlights

• We describe and quantify the global spread of the HIV-1 subtype B, the most prevalent in the Western World
• We show that the virus did not spread randomly by “diffusion” away from places with high virus burden
• Significant migration routes are consistent with geopolitical factors that affected human activities.
• Epidemic control policies should be global and incorporate political and socioeconomic factors
• Hidden underlying ecological pathways that define how pandemics of viral infections spread around the world.

## 1 Introduction

Human immunodeficiency virus (HIV) was discovered in the early 1980s (Barre-Sinoussi et al., 1983) when the virus had already established a pandemic. For at least three decades the epidemic in the Western World has been dominated by subtype B infections, as part of a sub-epidemic that traveled from Africa to United States through Haiti, and then to the rest of the world (Gilbert et al., 2007). Archival HIV sequences from the earliest known Haitian AIDS patients have helped science to understand early events in the spread of HIV (Gilbert et al., 2007). Genetic analysis of the epidemiologically homogenous epidemic in the United Kingdom (UK) among men having sex with men (MSM) has revealed multiple introductions of the virus to the country and distinct subepidemics (Hue et al., 2005). Within Europe it has been previously indicated that major tourist destinations have served as transmission outwards (Paraskevis et al., 2009), and also, as expected, neighboring countries are more likely to exchange viral strains than distant countries (Frentz et al., 2013). However, with the exception of local country-specific outbreaks and studies tracking the spread of the virus on a local scale, the global flow of subtype B during the last 30 years still remains to be charted.

With 0.3 mutations per genome per replication cycle in vitro (Mansky and Temin, 1995) and almost 40 mutations per genome per replication cycle in vivo (Cuevas et al., 2015) HIV-1 is among the fastest evolving human pathogens. Since the human host evolves much slower, pathogen-host evolutionary conflicts have not, yet visibly affected the host. HIV-1 has been infecting humans for less than 100 years, and mathematical models of the effect of HIV on human gene frequency indicate that it is unlikely to have shaped our evolution on these timescales (Cromer et al., 2010). On the other hand, large-scale human activity should be reflected in the global spread and evolutionary patterns of the virus (host-to-parasite) as it has been documented for other pathogens (Paraskevis et al., 2013). Available molecular sequences of the virus are an invaluable archive of the history of the epidemic. Quantifying the viral flows generates hypotheses to be tested and assessed on the potential effects of international public health measures.

HIV-1 has been extensively sequenced within part of the pol gene (protease, reverse transcriptase and integrase) mostly because this region harbors resistance mutations against the most commonly used antiretrovirals (protease, reverse transcriptase and integrase inhibitors) (Panel on Antiretroviral Guidelines for Adults and Adolescen, Rhee et al., 2003, Society, E.A.C., 2012, Vandamme et al., 2011). Despite the abundance of available viral sequences there is no large-scale systematic molecular surveillance of HIV-1 spread because most sequences are produced as part of routine clinical care and never published or deposited in public sequence databases. We thus set out to build a globally representative HIV-1 subtype B dataset of pol gene sequences from previous studies after a systematic search of the literature. Our aim is to clarify the global routes of the epidemic and understand how these were influenced by human activities over the last 50 years.

## 2 Materials and methods

### 2.1 Systematic collection of molecular sequences

#### 2.1.1 Non-European dataset

We collected non-European HIV-1 subtype B sequences, through a systematic bibliographic search in PubMed searching for molecular epidemiology or antiretroviral resistance studies for each country. We used the following keywords for the bibliographic search: “HIV-1”, “molecular epidemiology”, “resistance”, “subtype B” and “pol” in different combinations. We subsequently selected subtype B sequences from the retrieved studies to maximise representativeness and geographic coverage both globally and within each country. More details on the bibliographic search, collection and selection of sequences are available in Supplementary Information (SI).

#### 2.1.2 European dataset

The European dataset included sequences from two different sources: the Combined Analysis of Resistance Transmission over Time of Chronically and Acute Infected HIV Patients (CATCH) and the SPREAD (Strategy to Control SPREAD of HIV Drug Resistance) collaboration. The CATCH study included 2208 antiretroviral naïve individuals from 18 European countries and Israel during 1996–2002 (Wensing et al., 2005). Of those, 1601 were newly diagnosed cases and 607 were chronically infected patients, included in a retrospective setting. The prevalence of subtype B was 70% among the CATCH population (Wensing et al., 2005). Notably, although these data for 1996–2002 were retrospectively selected and pooled, they were originally collected as part of national surveillance studies of the transmission of drug resistance or as part of the standard clinical practice of baseline sequencing for all newly diagnosed cases in each participating center (Wensing et al., 2005). The SPREAD study included 4480 newly diagnosed patients sampled during 9/2002–12/2007 from 20 European countries and Israel. In the prospective setting a standardised sampling strategy was designed to include representative sampling from all countries (Vercauteren et al., 2009, Wensing et al., 2008). For the purpose of this study we included only those classified as subtype B from both the CATCH and the SPREAD studies.

In both studies all patients were older than 18 years and had not received antiretroviral therapy. More details on the sampling strategies have been published previously (Vercauteren et al., 2009, Wensing et al., 2005, Wensing et al., 2008). The sampling countries and the number of sequences per country after down-sampling are described in Table S1 in the supplemental material.

### 2.2 Details of the phylogeographic analyses

#### 2.2.1 Multiple sequence alignments and phylogenetic analysis

We aligned the HIV-1 sequences using ClustalW (version 1.82) and then manually corrected the alignment according to the encoded reading frame using MEGA5 (Hall, 2013). To avoid potential biases to our analysis resulting from convergent evolution due to selection of resistant isolates by antiretroviral treatment, we discarded codon positions known to confer antiretroviral resistance (PR: 30, 32, 33, 46, 47, 48, 50, 54, 76, 82, 84, 88, 90, and RT: 41, 62, 65, 67, 69, 70, 74, 75, 77, 100, 103, 106,108, 115, 116, 151, 181, 184, 188, 190, 210, 215, 219, 225, 236) (Lewis et al., 2008). The final alignment consisted of 222 codons and covers positions 2283–3191 of the HXB2 strain.

We estimated a phylogenetic tree from the nucleotide sequence alignment using ML under the general time-reversible (GTR) model of nucleotide substitution, including a G distributed rate of heterogeneity among sites as implemented in RAxML (Stamatakis, 2006, Stamatakis et al., 2008). We also estimated ML trees on 250 bootstrapped alignments to use on our subsequent phylogeographic analysis. We didn't use a higher number of bootstrap replicates because the calculations would be computationally expensive. Trees were rooted at the midpoint.

#### 2.2.2 Phylogeographic analyses

We used the bootstrap trees to estimate HIV-1 migration events among geographic regions with the parsimony approach described by Slatkin and Maddison, as implemented in PAUP* 4.0 (Slatkin and Maddison, 1989). Specifically, we assigned the tips of the inferred trees with a character according to the geographic origin of the patient (e.g. 0, 1, 2 for Austria, Belgium, Denmark, respectively, etc.). The viral migration events between different areas were estimated by DELTRAN optimization using only unambiguously reconstructed ancestral states. We provide more details in SI.

We were not able to implement a method that combines molecular clock and phylogeography such as the one used in BEAST; the large number of geographic regions along with the higher number of sequences make the analysis to be extremely computationally intensive (Lemey et al., 2009) and the sampling of the Bayesian Markov Chains did not converge (data not shown). On the other hand, a significant proportion of the sequences do not have isolation date, thus for a combined molecular clock-phylogeographic analysis we would have to discard a significant amount of our dataset reducing the representativeness of our sampling. Consequently, given that previous comparisons between parsimony and Bayesian analyses showed that a parsimony-based approach provides reasonably similar scenarios of geographic migration (Lemey et al., 2009) and have been widely used for similar analyses (Angelis et al., 2015, Paraskevis et al., 2009, Wallace et al., 2007), we chose the parsimony approach because it is feasible and provides reasonably accurate results by taking advantage of the most representative dataset.

#### 2.2.3 Steps of analysis

We performed the following phylogeographic analyses to identify viral transmission pathways:

1) Viral migration between large geographic areas. We grouped the viral strains in geographic areas, namely North America, Europe, Central & South America, Caribbean, Africa, Asia and Oceania. Since subtype B has a very low prevalence in many non-Western countries, the geographical regions of Africa, Asia and Oceania could not be further subdivided due to a lack of available sequences. We should note that in terms of the global HIV-1 epidemiology it would make sense to further split some geographic regions (similarly to North and South America), for example Africa could be split into North and sub-Saharan regions. However, subtype B has very low prevalence in non-Western countries resulting into low availability of sequences from these areas. Thus, we did not segregate further these regions, as it would then diminish statistical power. We provide detailed geographic sampling of the sequences in Table S1 and we pinpoint that spread inferences in low-prevalence geographic regions should be interpreted with caution not to generalize the result over the full geographic region, but to think of them as proxies of the global viral flow around the world (SI).
2) We repeated the above analysis by dividing Europe into Western and Central/Eastern Europe (WHO criteria) (see SI).
3) Viral migration between geographic areas (North America, Central & South America, Caribbean, Africa, Asia, Oceania) and European countries.
4) Viral migration within Europe. Only the European viral strains were used to infer migration routes among the European countries.

#### 2.2.4 Statistical phylogeography: taking into account sources of uncertainty in inferring migration events

We estimated which migration pathways were significantly different from the expected pathways under the null hypothesis of full geographic mixing (panmixis) of HIV-1 sequences. Thus, significance becomes independent from prevalence, and countries with a larger number of migration events can have non-significant migration if they have a high prevalence. Significance was established when the distribution of the migration events inferred from the 250 bootstrap trees was statistically different from the distribution of the events inferred from the same set of trees (*N* = 250) in each pathway after randomly reshuffling taxa at the tips as described previously (Angelis et al., 2015, Paraskevis et al., 2009). In a full geographic mixing case, an infected individual would have the same probability to transmit the HIV-1 to any other healthy individual, and all individuals are just as likely to share closely related viruses. Thus, a random reshuffling of taxa at the tips would simulate a tree inferred from such a population. The reshuffling was performed in the Mesquite program (Maddison and Maddison, 2015). We assessed equality of means between the observed and the expected migration events by means of one-sided Mann–Whitney test and adjusted the level of significance according to Bonferroni correction for multiple comparisons (for 32 localities we have 992 possible pathways/comparisons). We finally estimated the ratio between the observed and the expected value under the panmixis hypothesis (referred to as observed/expected ratio), which provides a quantitative metric of the relative spread of the virus between countries correcting for potential sampling bias due to unequal number of strains per country. Higher ratios suggest higher levels of viral exchange among locations. We randomly down-sampled (datasets I and II, see SI) and repeated all analyses (1) - (4) twice to assess robustness of the results. Only results from the first run are reported.

#### 2.2.5 Force of migration: a summary migration index

We summarize the exporting and importing migration for each geographic region using a new metric which we call Force of Migration (*F*_M_) and is defined as:

$$F_{M}=\frac{M_{E}\times E}{M_{I}\times I},$$

where *E* is the number of significantly exporting pathways that a region has, *M*_*E*_ is the total number of migration events from these exporting pathways, *I* is the number of significantly importing pathways that the region has and *M*_*I*_ is the total number of migration events from those importing pathways.

To create null distribution of migration indexes we have generated phylogeographic matrices from the randomly reshuffled (panmictic) phylogenies as described above (randomly-generated matrices). These panmictic matrices represent the case where the sequences included in the phylogenies do not come from a geographic structure, thus the observed migration can be simply explained by free random move within the same locality. To estimate the distribution of *F*_M_ we compare each of the bootstrap-generated migration matrices with one randomly selected matrix from the set of the randomly-generated matrices. If a cell (migration counts) of the bootstrap-generated matrix contains more migration events than the respective cell of the randomly-generated matrix we consider it to be significant and include it in the calculation of the *F*_M_. We thus obtain 250 *F*_M_ values (for each geographic region), which correspond to the distribution of the observed *F*_M_. To generate a null distribution of *F*_M_ values we compare each one of randomly-generated matrices against a randomly chosen matrix from the rest of the randomly-generated matrices. We thus obtain 250 *F*_M_ values (for each geographic region), which correspond to the null (expected) distribution of *F*_M_. We test if the observed values of *F*_M_ differ significantly from the expected distribution of *F*_M_ by means of the Mann–Whitney test taking into account multiple comparisons (Bonferroni correction). We use this metric to classify whether a geographical unit is actively spreading (“outward”) or passively receiving (“inward”) the subtype B epidemic.

#### 2.2.6 Non-European connectivity index

To estimate if a Western European country is more connected with non-European regions than expected we calculate for each Western European country an out-of-Europe export index as follows:

$$C_{n}=\frac{\left(\text{total}\;\text{number}\;\text{of}\;\text{significant}\;\text{migration}\;\text{events}\;\mathrm{to}\;\mathrm{non}‐\text{European}\;\text{targets}\right)}{\left(\text{total}\;\text{number}\;\text{of}\;\text{significant}\;\text{migration}\;\text{events}\;\mathrm{to}\;\text{European}\;\text{targets}\right)}$$

We calculate this index for the observed and the expected (bootstrapped) phylogeographies and then we statistically test using a Mann–Whitney test if the observed index is higher than the expected (this being equivalent to testing whether the ratio of the observed/expected is higher than 1). For simplicity we summarized the propensity to export more by producing the ratio of the observed *C*_*n*_ versus the expected *C*_*n*_ (Fig. S4 in the supplemental material); ratio higher than 1 means the country exports more to non-European regions than to Europeans than randomly expected.

### 2.3 Molecular clock analysis

We estimated the time to Most Recent Common Ancestor (tMRCA) for five clusters of sequences from Central and Eastern Europe including reference sequences with known sampling dates. We focused on monophyletic clusters from C.E. European countries were geographically defined phylogenetic clusters including ≥ 75% of sequences from C.E. Europe. These clusters were selected in order to estimate the tMRCA of the regional epidemics spreading in this area. To increase the sampling window of sequences from C.E. Europe, we included 9 sequences sampled from North America, Europe and Asia (sampling period between 1983 and 2004). We used a Bayesian approach as implemented in BEAST version 1.8.0 (Drummond and Rambaut, 2007) with a GTR + G model of nucleotide substitution. We used the uncorrelated lognormal relaxed clock model (Drummond et al., 2006) with TipDates and a non-parametric coalescent approach (Bayesian skyline) (Drummond and Rambaut, 2007). Markov chain Monte Carlo (MCMC) was run 2 times for each cluster for 30 × 10^6^ generations with a burn in of 30 × 10^5^ sampling every 1000 iterations. For the largest cluster (*n* = 230 sequences) MCMC was run for 90 × 10^6^ generations with a burn in of 20 × 10^6^, sampling every 1000 generations. Convergence was assessed using Tracer v1.5 (Rambaut et al., 2013-12-11) and an Estimated Sample Size (ESS) larger than 200. The consensus tree for each run was estimated by the TreeAnnotator program (Drummond and Rambaut, 2007).

## 3 Results

We first use the nucleotide alignment to reconstruct the phylogenetic relationships among viral strains. We take into account phylogenetic uncertainty by estimating several phylogenetic trees via maximum likelihood (ML) method using bootstrap resampling. We then assign geographic sampling information at the tips of the bootstrap trees and reconstruct the past movement of the viral strains across the geographic regions by estimating the number and direction of viral migration events using parsimony (Slatkin and Maddison, 1989). We then use this information to identify viral migration routes and test for their statistical significance. Different geographical grouping strategies of the viral strains are used in order to track the viral spread in different spatial scales and to assess robustness of the inferred transmission patterns. We also introduce new metrics to classify specific geographic regions into “outward” (regions where HIV mostly departed from), “inward” (regions that mostly received HIV) or “isolated” (regions where HIV exchange with other regions was much lower) and explore viral connectivity links among particular areas. Finally we test our results for epidemiological consistency and date sampling bias.

### 3.1 Source of data

We pooled HIV-1 pol gene sequences from three sources, two European cohort sequence databases (see European dataset in Materials and methods) and a dataset with publicly available sequences, which we selected through a systematic search of the literature (see Non-European dataset in Materials and methods) (Vercauteren et al., 2009, Wensing et al., 2005, Wensing et al., 2008). In total we collected 10,078 sequences from 78 countries representing the vast majority of countries that are affected by the subtype B global pandemic (Table S4).

### 3.2 Patterns of regional clustering inferred by phylogenetic analysis

**Fig. 1:**
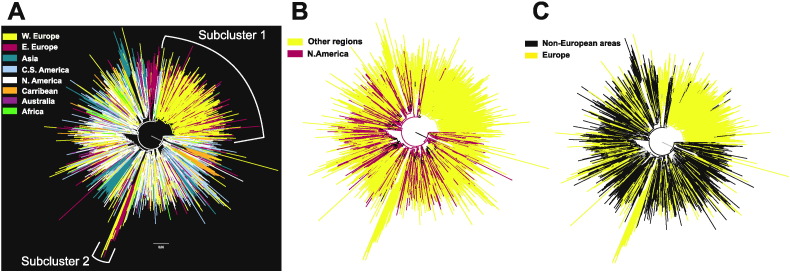
ML phylogeographic tree showing viral clades in different colors according to sampling area. A: Clades from eight different geographic areas are highlighted. B: Clades are separated into North American and other regions and C: clades are separated into European and non-European. W. Europe: Western Europe, C.E. Europe: Central/Eastern Europe, C.S.·America: Central and South America, N. America: North America.

We used our subtype B alignment (Dataset I, Table S1) to estimate a ML phylogenetic tree and we colored its viral clades in different colors according to sampling location in order to infer phylogenetic relationships among viral strains from different sampling locations. The global colored phylogeographic trees show that European strains tend to cluster together, whereas North American strains are very dispersed among the global genetic diversity (Fig. 1A). More specifically, 1787 HIV-1 sequences, that is 44% of the total European sampled population (*N* = 4020) (Fig. 1C), were included in a single clade; 71% of the sequences in this large clade were sampled from Western Europe (*N* = 1274) (Fig. 1A, Subcluster 1). We also detected another mainly European clade, which included strains from both Western and Central/Eastern Europe (Fig. 1A, Subcluster 2). Asian and Caribbean sequences showed clustering patterns and formed several clades in a way similar to Europe (Fig. 1A). In contrast, North and Central & South American lineages were widely distributed across the global phylogeny suggesting that HIV-1 spread is higher between these areas and the rest of the world. North American strains tend to be closer to the root than groups of sequences found in multiple other areas (i.e. shown in white and red color in Fig. 1A and B, respectively). In the phylogeographic tree (Fig. 1C) with isolates categorized in European and non-European groups we see that European specific clades seem to be nested within non-European founders.

A phylogeographic tree might only be suggestive of global migration patterns and can provide only limited quantitative information of viral spread among countries. Crucially, statistical support of clades with bootstrap values in these trees is expected to be low due to the high number of closely related sequences included in the analyses (Sanderson and Wojciechowski, 2000), which does not allow for inference of source-sink patterns. Thus, in order to evaluate viral spread we use a statistical phylogeography approach, which provides a formal framework to evaluate significance of viral migration by quantifying viral exchanges.

### 3.3 Tracing the spread of HIV-1 subtype B

In the following sections we identify migration patterns of subtype B around the globe by means of statistical phylogeography. To control for potential sampling bias and quantify spread at different geographical scales, we performed analyses with four levels of geographical segregation. We grouped viral strains according to: (1) large geographical regions Europe, North America, Central/South America, Caribbean, Africa, Asia and Oceania (WHO criteria), (2) as in (1) but splitting Europe into Central/East and Western Europe and (3) as in (1) but splitting Europe into countries and (4) grouping only European viral strains by country to estimate viral migration only within Europe.

First, we comment on the migration routes arising from the statistically significant migration events and then we test for epidemiological consistency and robustness of results. Quantitative details (number of migration events and statistical significance of pathways) are provided in Table 1, Table 2 and Tables S2 and S3 in the supplemental material.

#### 3.3.1 Global spread

**Fig. 2:**
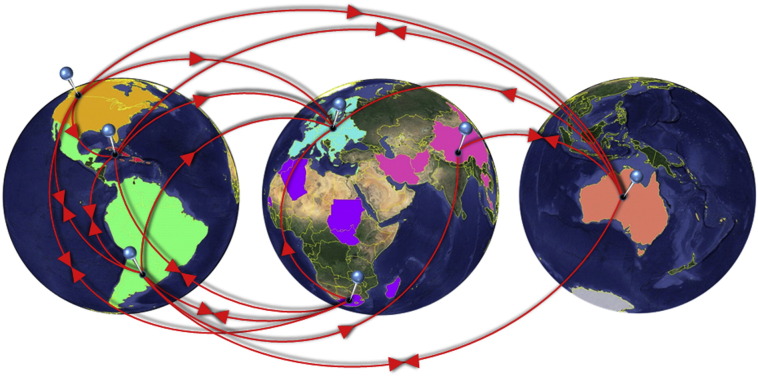
Global migration patterns of HIV-1 subtype B estimated by statistical phylogeography under the geographical grouping strategy 1. Colors indicate different geographic regions (highlighted countries) from which HIV-1 sequences were available. Pins represent different geographic regions (group of highlighted countries) and arrows indicate the direction of subtype B spread.

Considering the global migration of HIV-1 subtype B between large geographical regions. Europe was not a significant “outward” of subtype B towards other regions (Table 1 – “Europe” row has no statistically significant outgoing events to any region). It receives infections from all other regions except Asia (Table 1 – “Europe” column). The significant pathways towards Europe are also supported by high ratios of observed over expected events indicating high levels of viral importation. The American geographical regions were “outwards” of infections exported to the rest of the world. North America was an “outward” of viral migration to all regions except Asia (see Table 1 – “N. America” row). Central/South America and the Caribbean were also found as direct “outward” for viral spread to other areas; however the two pathways with the highest statistical significance out of these regions were from Central/South America to North America and from the Caribbean to Europe. Viral importation to North America took place mostly from Central/South America (supplemental information). Asia is the most isolated area with the fewest significant incoming and outgoing destinations; it has connections only with Oceania and Central/South America. The viral spread among those large geographic regions is illustrated in Fig. 2.

#### 3.3.2 Different roles for Western and Central/Eastern Europe

Since Western and Central/Eastern Europe have quite distinct epidemic histories, we repeated the statistical phylogeographic analysis after splitting Europe accordingly (Fig. S1). Comparing this new analysis with the one where Europe was not split, we see that the viral spread remained robust. We found no statistically significant viral migration towards C.E. Europe (Table 2 – “C.E. Europe” column), but instead some significant spread from C.E. Europe to Western Europe (Table 2 – “C.E. Europe” row). Thus, the high incoming viral spread towards Europe (Table 1) observed in the global analysis is due to incoming spread particularly to Western Europe rather than to the whole continent; while C.E. Europe seems to be isolated. Indeed, the phylogeographic tree suggests that C.E. European strains seem to accumulate in well-formed distinct clades (Figs. 1A, S2 in the supplemental material), a pattern which suggests that they are more related with each other than with strains isolated in other parts of the globe.

#### 3.3.3 Viral spread among European countries

We then explored viral spread of the different European countries separately in order to detect a finer pattern of viral global spread, as was the case for the West and C.E. Europe above. Results are in accordance with the above-mentioned pattern and indicate that C.E. European countries seem to be more isolated (Table S2 in the supplemental material, supplemental information). Some C.E. European countries such as Albania, Romania and Belarus had fewer significant migratory targets. More specifically all countries in C.E. Europe were exchanging viruses with a smaller number of countries (i.e. 1–8) in comparison to Western Europe (i.e. 2–18) except for Poland and Czech Republic/Slovakia for which we found a larger number of connecting countries (5–15) (Fig. S3, Table S2 in the supplemental material).

Concerning viral spread among the European countries we find evidence that they were highly interconnected (Fig. S3B, supplemental information). Viral spread within Europe seems to be high. Some countries like Portugal, Spain and Germany exchange HIV with many other countries (Table S3 in the supplemental material). Within Western Europe the most connected country seems to be Spain, both quantitatively in migration events, and also in the number of countries with significant exchanges of infection (Fig. S3A,B).

#### 3.3.4 Quantification of viral migration: “outwards”, “inwards” and “isolated” regions

We introduced a simple metric (Force of Migration or *F*_M_, see Materials and methods) to quantify if a geographical unit (whether it is a region or a country) is actively spreading or passively receiving viral migrations. *F*_M_ is larger for geographic units that have more exporting targets and associated exporting migration events, and smaller for geographic units that have more importing targets and associated migration events. We test the statistical significance (corrected for multiple comparisons with the Bonferroni formula) of *F*_M_ for each geographic unit against a simulated distribution assuming random exporting and importing events. We categorize a geographical unit as an “outward” if it spreads viral strains more than expected, “inward” if it receives viral strains more than expected and “isolated” if it is exchanging viral strains with other regions less than expected. We comment on the most striking findings of *F*_M_ from each of the above-mentioned geographic segregations.

In the analysis where we considered Europe to be a single geographic region, N. America had (median) *F*_M_ = 61.59 which is 113 times higher than what expected by chance (expected *F*_M_ = 0.543, *p* < 0.001), so it is an “outward” of viral migration, Europe is a sink *F*_M_ = 0 much less than the expected value *F*_M_ = 26.54, *p* < 0.001. When we separate Europe into West and Central/East, N. America still remains an “outward” (*F*_M_ = 58.67, 45 times higher, *p* < 0.001), Western Europe remains a sink (*F*_M_ = 0, expected *F*_M_ = 12.62, *p* < 0.001), but C.E. Europe is “isolated” with no significant exporting or importing viral migration. We note that *F*_M_ cannot be determined for “isolated” regions due to zeros in both nominator and denominator, but the statistical significance for being “isolated” can be estimated by comparing the distribution of the non-significant events in the observed against the simulated trees. We found that 247 out of 250 trees showed no significant migrations in the observed dataset (i.e. the phylogenetic trees of the bootstrapped alignment), while only 19 out of 250 simulated trees had no significant events (*p* < 0.001, significant for multiple testing).

To address whether the above pattern for N. America is biased by earlier sampling dates for N. American strains, we repeated the same analysis (with Europe as a single region) on a dataset where we randomly subsampled sequences to keep the ratio of European to N. American isolates at 2:1 for each sampling year (i.e. to keep roughly the same overall ratio as in the large dataset). The N. American *F*_M_ was smaller than when we do not account for sampling date, but still much higher than expected (28 times, *p* < 0.001); same for the European *F*_M_ = 0 (again much lower than expected, *p* < 0.001). Thus, the observed pattern of N. America being an outward and Europe being an inward is robust with respect to sampling date.

### 3.4 Exceptions to the Western European “sink”

Since Western Europe was found to be a sink, we analysed whether there are any countries within Western Europe deviated from this pattern i.e. are more connected to non-European countries. We made an index that is equal to the ratio of the observed total migration events (importing and exporting) to non-European divided by the total migration events to European regions. This ratio if larger than 1 indicates that a country is more connected to non-European countries than to European ones and can be tested for its statistical significance against the ratio expected by chance with a standard non-parametric test (Mann–Whitney test to compare observed against expected). Three countries were found to have a large significant ratio, more specifically the United Kingdom (ratio = 1.8), Switzerland (ratio = 1.6) and France (ratio = 1.5) (all having *p* < 0.001, significant for multiple comparisons) (Fig. S4).

### 3.5 Dating the establishment of epidemics in the C.E. European “isolation”

Even though C.E. Europe was found to be “isolated” it must have been seeded with subtype B at some point in time. We, thus, performed phylodynamic analysis in five major monophyletic clusters from this region. These clusters consisted of 10, 21, 38, 67 and 230 sequences from Slovenia (cluster I), Slovenia/Bulgaria (cluster II), Slovenia (cluster III), Romania (cluster IV), and Poland/Bulgaria/Ukraine (cluster V), respectively. The estimated time of the most recent common ancestor (tMRCA) corresponding approximately to the time of the origin of HIV-1 epidemic in these areas ranged between 1987 and 2001 (median estimates). Cluster I was estimated to be the most recent, with estimated tMRCA in 2001 (median value, 95% Higher Posterior Density HPD: 1999–2003). For clusters II and III tMRCA was estimated in 1989 (95% HPD: 1984–1993) and 1996 (95% HPD: 1992–1999), respectively. For the largest one (cluster V) including HIV-1 sequences from Poland, Bulgaria and Ukraine the tMRCA was in 1987 (95% HPD: 1982–1990). Finally for cluster IV from Romania the date of the most recent common ancestor was previously estimated in 1991 (95%HPD: 1983–1999) (Stanojevic et al., 2012).

### 3.6 Viral migration is epidemiologically consistent

As a final step of our analyses we wished to evaluate whether viral migration, as we quantified it with statistical phylogeography, is consistent with known epidemiologic surveillance. Standard mathematical models predict that transmission is higher in populations with more infected individuals suggesting that countries with higher number of infected persons should drive viral migration to other countries or, in other words, be more prone to a spillover effect (Anderson and May, 1991, Grassly and Fraser, 2008, Keeling and Rohani, 2008). Thus, we would expect countries with higher number of prevalent cases to be more likely to provide spillovers to other countries. To examine whether our analyses is consistent with this expectation, we estimated the country-specific number of HIV subtype B infections by multiplying the total number of people living with HIV-1 per country until 2011, based on the UNAIDS figures (www.unaids.org), by the percentage of subtype B in each country (Abecasis et al., 2013, Avi et al., 2009, Balode et al., 2012, Ciccozzi et al., 2005, Ivanov et al., 2013, Saad et al., 2006, Stanojevic et al., 2012, Ustina et al., 2001). We then examined the association between the number of HIV subtype B infections per country/region and the number of countries that each country/region exported viral strains in analysis (4) by running a regression analysis. We log-transformed the number of subtype B infections per country/region because its distribution among countries/regions is skewed; 6 out of 24 countries/regions with the most HIV-1 subtype B prevalent cases (these are UK, France, Italy/Ireland, Spain, Ukraine, Germany) account for more than 80% of the subtype B infections in Europe as a typical long-tail distribution.

**Fig. 3:**
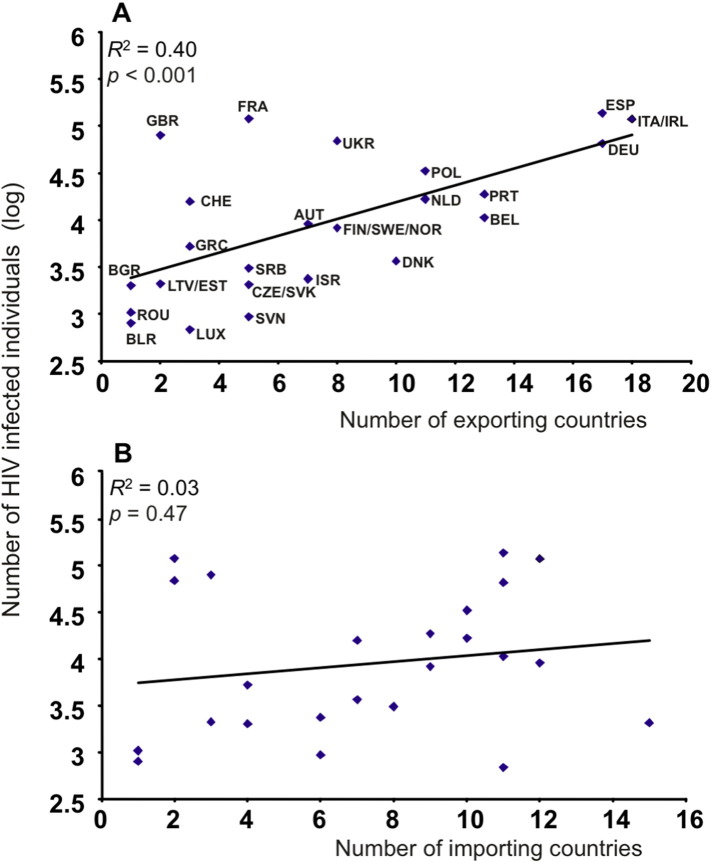
A: Scatter plot of the total number of HIV-1 subtype B infected individuals in log scale per country/region versus the number of exporting pathways for each country/region. The solid line is the fitted regression line. *R*^2^ is the coefficient of determination and *p* is the *p*-value of the regression model. Country names are shown only for the statistically significant regression in ISO (International Organization for Standardization) three-letter codes; ALB: Albania, AUT: Austria, BEL: Belgium, BGR: Bulgaria, BLR: Belarus, CHE: Switzerland, CZE: Czech Republic, DEU: Germany, DNK: Denmark, ESP: Spain, EST: Estonia, FIN: Finland, FRA: France, GBR: United Kingdom, GRC: Greece, IRL: Ireland, ISR: Israel, ITA: Italy, LTV: Latvia, LUX: Luxembourg, NLD: Netherlands, NOR: Norway, POL: Poland, PRT: Portugal, ROU: Romania, SRB: Serbia, SVK: Slovakia, SVN: Slovenia, SWE: Sweden, UKR: Ukraine. B: Same as A, but with the number of importing pathways for each country/region.

The number of exporting countries significantly correlates with the number of subtype B infections in the outward country (*R*^2^ = 0.40, *p* < 0.001 and *p* = 0.002 using the nonparametric Spearman correlation coefficient), suggesting (as expected) that areas with high number of HIV prevalent cases are more likely to export infections to other countries (Fig. 3A). The correlation is robust against the number of sequences sampled per country (i.e. it is not the result of including more sequences from countries with higher prevalence). Germany, Italy/Ireland and Spain have the higher number of exporting targets but also account for more than 44% of the HIV-1 subtype B infections in Europe. To test if these countries are influential points for the correlation, we removed them from the regression analysis and the relationship between number of exporting countries and number of prevalent cases within the outward country is weaker, but still highly significant (*R*^2^ = 0.26, *p* = 0.015 and using Spearman *r*_*s*_ *=* 0.58, *p* = 0.004). To take into account the dissimilarities between Eastern and Western Europe we add a dummy variable in the above regression model indicating if the country belongs to Central/Eastern or Western Europe (according to WHO criteria). The analysis suggested that the number of prevalent cases indeed remained a significant predictor (*p* = 0.004). Crucially, the scatter plot (Fig. 3A) suggested that UK and France lie far from the regression line, which means that they do not provide as many spillovers within Europe as it would be expected from their high number of prevalent cases. Removal of UK and France from the analysis dramatically improves the correlation (*R*^2^ = 0.69, *p* < 0.001 or Spearman *r*_*s*_ = 0.83, *p* < 0.001) suggesting that UK and France had a different spillover pattern than the rest of the European countries. We found no significant correlation between the number of prevalent cases within a country/region and the number of countries which are source of viral introduction to that country/region (*R*^2^ = 0.03, *p* = 0.47, Fig. 3B).

## 4 Discussion

Our study describes the global pattern of HIV-1 migration across the Western Hemisphere. Molecular methods have been extensively used for the characterization of the HIV-1 migration; however, the global routes of epidemic migration remain uncovered. In our study, we used a global dataset collected after a systematic bibliographic search, and the inference of cross-border transmissions was based on statistical phylogeographic approach as described previously (Angelis et al., 2015, Cottam et al., 2008, Magiorkinis et al., 2009, Paraskevis et al., 2009, Paraskevis et al., 2013, Wallace et al., 2007). Moreover we developed a new metric for the classification of geographic areas as “outward”, “inward” and “isolated” according to their estimated pattern of incoming or outgoing viral spread pattern.

We found that the American continent and the Caribbean acted as “outwards” for the Western epidemic not only at the initial random migration event (Gilbert et al., 2007), but also through constant subsequent spread to the rest of the world. The striking role of America in disseminating subtype B infections was probably a consequence of the early introduction and propagation of this clade in North America remaining silent for almost a decade (Gilbert et al., 2007).

In striking contrast, subtype B infections in Europe, and specifically in Western Europe, were introduced as a result of multiple introductions from different geographic areas. This is in accordance with previous studies suggesting that many of the earliest HIV(+) cases in Europe among the MSM had links to N. America (Pinching, 1984), and also that independent introductions from N. America to Europe occurred among MSM in the UK (Hue et al., 2005) and in injecting drug users in Northern Europe (Lukashov et al., 1996). Our study adds a piece in the puzzle with regard to the HIV-1 global migration patterns. The HIV-1 epidemic was documented first among the MSM in the United States, in early eighties, as the result of an early introduction from the Haiti (Gilbert et al., 2007). Subsequently, the epidemic spread to Europe and other areas in the Western Hemisphere, however until now the question with regard to the patterns of cross-continental transmissions of the currently circulating strains remains unanswered. Our study describes the distinct role of different geographic areas in driving the Western epidemic, highlighting that the role of Europe for this subepidemic with respect to the rest of the globe was secondary and the incoming infections spread mainly among regional populations. Additionally we show that viral spread to Northern America occurred mostly from Central/South America; Asia on the other hand, was the most isolated area. The former can be explained by cross-border movements of people across the American continent, while the “isolated” nature of subtype B epidemic in Asia, previously described as the monophyletic clade “B′”, is probably due to the local spread of this clade among the intravenous drugs users (IDU) and former plasma donors (FPDs) in Asia (Li et al., 2010).

After separating Europe we show that C.E. Europe did not import more than expected from Western Europe even though Western Europe had historically higher levels of subtype B infection which might be explained due to the historically low population mobility among these regions of Europe. We estimated the date of this clear phylogeographic segregation of West and C.E. European strains by means of molecular clock analysis to be roughly between late 80s and late 90s, which is around the separation of the post-Soviet countries. With regard to C.E. Europe, the viral spread pattern can be characterized as “isolated”. A similar pattern has also been detected for the subtype A spreading among the local IDU population (A_FSU_) in Easter Europe (Bobkov et al., 1998, Thomson et al., 2009), showing almost no links with other geographic areas. The similarity in the “isolated” pattern of viral migration in Eastern Europe for both subtypes A and B, although that they have spread through different routes (parenteral and sexual transmissions), strongly suggest that factors like limited population mobility and high risk behavior of drug injectors have played a significant role in shaping the characteristics of HIV-1 epidemic spread in Eastern Europe that remains isolated. We also see that C.E Europe provided sources of viral migration towards Western Europe; specifically Poland and the Czech Republic showed more connectivity to Western European countries, than the rest of the C.E. European countries (Figs. S5 and S6 in the supplemental material). Poland was the first country that loosened its ties from the Eastern European block, which could probably explain its closer ties with Western Europe than the other former communist countries in C.E. Europe. Similarly Czech Republic had always been in closer connection to Western Europe in comparison to other Eastern European countries mainly due to its central position in Europe. We, thus, suggest that the viral migration pattern between Western and C.E. Europe can be also explained as a result of the separation of these two parts of Europe from the end of World War II in 1947 till the end of Cold War in 1989 (Hansen, 2002). Soon after the split of the Soviet Union human migrations from Eastern European countries to Western Europe was notable, and this is mirrored by the recent introduction of Eastern European isolates in Western European Countries.

It is noteworthy that the highest spread between any European country and non-European regions was observed for UK, Switzerland and France (Fig. S4 in the supplemental material). UK and France show similar patterns with connections to Americas and Africa and we have also found that for the correlation between number of prevalent cases and number of significant migratory routes, UK and France had a striking deviation. This is in accordance with our findings that both these countries provide major sources of viral spread from outside Europe (North, Central & South America and Caribbean), suggesting the distinct nature of these countries with regards to epidemic spread in Europe. UK and France are two of the largest countries in Europe with significant social and economic links across the globe that may explain their central role as epidemic importers.

Switzerland had also high connectivity with non-European countries, but shows a different pattern than UK and France. It did not deviate as strikingly as UK and France from the regression line between the number of prevalent cases and the number of significant migratory routes within Europe (Fig. 3A). Our findings are in accordance with a previous study suggesting that sequences from MSM clustered within local transmission networks at low proportions, suggesting multiple introductions from abroad (Kouyos et al., 2010).

Considering HIV spread among European countries, the seemingly most influential (both as exporter and importer) in accordance with previous studies (Paraskevis et al., 2009) is Spain (Fig. S3) having many significant viral exchange routes with other European countries. Several factors might have contributed to this, first Spain has the highest number of HIV-1 subtype B infections, thus is more likely to spill-over to other countries. Its high connectivity might be connected with the fact that within the last part of the 20th century, although the unemployment rate has been continuously high, Spain experienced a rapid migration turnover from a traditional exporter to a significant immigration destination (Bentolila et al., 1990). Being also among the most popular tourist destinations is likely to have contributed to the observed pattern (Paraskevis et al., 2009).

Notably, we found that the exporting viral spread, as measured by the number of exporting countries within Europe, correlates with the number of infections due to subtype B in the source country. We, thus, suggest that in general higher prevalent countries are more likely to act as sources for cross-border infections within Europe.

Since our study is retrospective, it is unlikely to provide strong evidence for causality in viral migration. Based on the inferred global pattern of HIV-1 subtype B viral spread, we may hypothesize that the outgoing viral spread for N. America and the Caribbean was probably due to cross-border transmissions occurred at the early stage of the epidemic when it was silent; a hypothesis further supported by the finding that viral lineages from N. America branched close to root of the tree. Central & South America show the most extensive network for outgoing spread probably due to immigration originated from these areas. In sticking contrast, Europe was an inward over the course of the epidemic, suggesting significant domestic migration for this subepidemic. Finally, Asia was the most isolated due to specific way of HIV-1 subtype B migration among the local networks of IDU and FPDs. Therefore the global spread of subtype B was not random but differs significantly across the continents.

Many mathematical models can predict the potential of pathogens to successfully establish an epidemic based on transmission parameters (Anderson and May, 1991, Grassly and Fraser, 2008, Keeling and Rohani, 2008). Theory suggests that epidemics during their early stages are sensitive to stochastic effects due to the small number of infected individuals (Bailey, 1953); presumably the route taken by the initial migration of HIV-1 from Africa to the US is the initial less predictable stochastic event. As pathogens become more prevalent (i.e. infect a larger proportion of the population) the overall dynamics operate in approximately deterministic way (Whittle, 1955); accordingly we show that European countries with higher HIV subtype B burden are more likely to spill infections over other countries. We, thus, show that the global viral phylogeography of HIV subtype B was not random and suggest that, since major landmarks of the last part of the 20th century influence human (hence virus) mobility, the virus spread around the globe is largely the result of natural virus-host ecological dynamics. Thus, our study provides working hypotheses as to how socio-economic circumstances influence the human-virus ecological dynamics and advocates in support of scaling-up collaboration of health system for preventing the spread of chronic viral infections.

## 5 Limitations

One major limitation of our study is that the collection of the samples/molecular sequences has not been performed under a common framework, which might make our analyses prone to sampling bias. To the best of our knowledge this drawback is present in all the phylogeographic studies published for HIV-1 up to date, as a systematic collection of sequences on large geographic areas has not been performed.

However, here for the first time we have systematically approached the sampling bias problem in multiple levels including our design and analyses by: 1) using sequences collected within well-defined cohorts allowing for uniformity of inclusion criteria at least within Europe, 2) collecting sequences with a meta-analyses approach rather than sequence database download for the non-European datasets, again allowing for more uniformity of inclusion criteria, and 3) analysing 2 sub-datasets to show robustness of results against potential sampling bias. We find no evidence of sampling bias in our analysis and we argue that includes the most representative and systematically composed sequence dataset that has been used for phylogeography studies of HIV-1 up to date.

The following are the supplementary data related to this article.

Supplementary material. Image 1

Supplementary figures. Image 2

Supplemental Table 1 Number of sequences per country. The Initial Dataset describes the number of sequences per country downloaded after the bibliographic search. Datasets I and II were used in the analyses. Supplemental Table 1

Supplemental Table 2 A. Means of observed migration events across all bootstrap trees between large geographic regions and European countries.
B. Ratio of mean of observed over mean of expected migration events between large geographic regions and European countries. Supplemental Table 2

Supplemental Table 3 A. Means of observed migration events across all bootstrap trees between the European countries.
B. Ratio of mean of observed over mean of expected migration events between the European countries. Supplemental Table 3

Supplemental Table 4 List of studies fulfilling selection criteria. Supplemental Table 4

## Funding information

This work was supported by the European Community's Seventh Framework Programme (FP7/2007–2013, under the project Collaborative HIV and Anti-HIV Drug Resistance Network; project number: 223131), the Medical Research Council (to G. M.; grant nr MR/K010565/1), the National Institute of Health (to S.R.) [grant nr DP1DA034989] and the AIDS Reference Laboratory of Leuven (which receives support from the Belgian Ministry of Social Affairs through the Fonds voor Wetenschappelijk Onderzoek–Flanders to K. T. [grant nr PDO/11 and G069214N]).

## Potential conflicts of interest

All authors declare that they have no conflicts of interest.

## Figures and Tables

**Table 1:** Mean of observed migration events (1st row) across all bootstrap trees for each pathway and ratios of mean of observed over mean of expected events (2nd row, in italics) under geographic grouping strategy 1.

	Importing to							
		N.·America	C.S. America	Caribbean	Africa	Asia	Oceania	Europe
Exporting from	N.·America		**175.27**	**40.03**	**28.16**	38.79	**30.81**	**291.17**
			***3.82***	***1.85***	***3.01***	*0.89*	***2.49***	***4.16***
	C.S.·America	**25.24**		**7.88**	**3.70**	**8.29**	**5.50**	**42.22**
		***2.13***		***2.36***	***2.23***	***1.20***	***2.98***	***5.95***
	Caribbean	1.48	**2.15**		**1.37**	0.78	**0.84**	**5.06**
		*0.75*	***1.72***		***5.80***	*0.70*	***2.43***	***6.05***
	Africa	**0.57**	**0.57**	**0.25**		0.16	0.10	**0.75**
		***1.84***	***2.25***	***2.25***		*0.85*	*1.73*	***8.13***
	Asia	1.90	2.67	1.20	1.01		**3.06**	3.67
		*0.19*	*0.44*	*0.41*	*0.78*		***1.74***	*0.67*
	Oceania	0.48	**0.65**	**0.50**	0.07	**1.14**		**1.19**
		*0.95*	***1.76***	***2.91***	*1.31*	***2.94***		***5.73***
	Europe	147.67	100.34	24.36	28.04	19.46	21.76	
		*0.18*	*0.23*	*0.12*	*0.33*	*0.05*	*0.20*	

Note. Cells in bold indicate statistically significant pathways (compared to the null-hypothesis of panmixis) after Bonferroni correction for multiple comparisons. N.·America: North America, C.S. America: Central & South America.

**Table 2:** Means of observed migration events (1st row) across all bootstrap trees for each pathway and ratios of mean of observed over mean of expected events (2nd row, in italics) under geographic grouping strategy 2.

	Importing to								
		N.·America	C.S. America	Caribbean	Africa	Asia	Oceania	C.E. Europe	W. Europe
Exporting from	N.·America		**186.06**	**42.37**	**29.95**	41.35	**32.81**	54.13	**287.58**
			***2.56***	***1.20***	***2.03***	*0.60*	***1.65***	*0.61*	***2.51***
	C.S. America	**25.79**		**8.12**	**3.97**	8.69	**5.70**	7.90	**41.54**
		***1.67***		***1.70***	***1.87***	*0.90*	***1.97***	*0.65*	***3.62***
	Caribbean	1.43	2.17		**1.38**	0.78	**0.85**	0.29	**5.04**
		*0.60*	*1.21*		***3.22***	*0.48*	***1.71***	*0.15*	***3.42***
	Africa	**0.57**	**0.62**	**0.27**		0.16	0.11	0.29	**0.76**
		***1.74***	***2.74***	***2.23***		*0.76*	*2.25*	*1.06*	***4.30***
	Asia	1.86	2.68	1.22	1.02		3.08	0.30	3.64
		*1.40*	*0.31*	*0.29*	*0.55*		*1.21*	*0.03*	*0.37*
	Oceania	0.46	**0.66**	**0.52**	0.08	**1.16**		0.45	**1.16**
		*0.68*	***1.43***	***2.60***	*1.36*	***2.56***		*0.88*	***2.85***
	C.E. Europe	1.92	1.47	1.22	0.24	0.57	0.49		**25.88**
		*0.07*	*0.08*	*0.14*	*0.06*	*0.03*	*0.09*		***1.14***
	W. Europe	109.53	78.35	18.75	23.51	14.62	16.30	162.36	
		*0.22*	*0.28*	*0.14*	*0.42*	*0.06*	*0.22*	*0.47*	

Note. Cells in bold indicate statistically significant pathways (compared to the null-hypothesis of panmixis) after Bonferroni correction for multiple comparisons. N.·America: North America, C.S. America: Central & South America, C.E. Europe: Central/Eastern Europe, W. Europe: Western Europe.
