# Supplementary material for: The global spread of HIV-1 subtype B epidemic
Source: Infect Genet Evol. 2016 Dec;46:169–79. doi: 10.1016/j.meegid.2016.05.041 (PMC5157885; doi:10.1016/j.meegid.2016.05.041)
Supplement: Supplementary file 1 — Supplementary material. [file mmc1.pdf]

The global spread of HIV-1 subtype B epidemic

Materials and Methods

Systematic collection of molecular sequences

Non-European dataset

We selected subtype B sequences from the retrieved studies to maximise representativeness and geographic coverage both globally and within each country. The sampling criteria were: i) in cases where more than one study was available for one country, we included only those sequences isolated from different areas of that country, ii) in cases where the sampling areas were not described in the studies from the same country, to avoid redundant sequences, we only included sequences from the largest study, iii) similarly for studies performed at the same centres or cities, iv) from longitudinal studies concerning mainly resistance to antiretroviral therapy, we included only the oldest available sequence per patient, and v) we excluded studies concerning mother to child transmission. No selection criteria were set with respect to treatment history of patients. The collected studies fulfilling our selection criteria are shown in Table S4.

We downloaded the HIV-1 sequences from the selected studies using either the NCBI nucleotide sequence database or the Los Alamos HIV sequence database (1). We subtyped sequences without subtype designation with the COMET tool (2) and further confirmed with phylogenetic analyses with subtype B reference strains as previously described (3). For countries having a much larger number of sequences than the rest, we randomly down-sampled in datasets I and II (Table S1) in order to avoid overepresentation from specific regions (see Steps of analysis, below).

Phylogeographic analyses

The algorithm assigns "ancestral" states to the internal nodes using the criterion of parsimony, thus reconstructing ancestral geographic regions. Parsimony reconstruction minimizes the total number of ancestral state changes across the nodes on the tree. Ancestral state changes occur at nodes when branches descending from those nodes lead to nodes or tips with different ancestral states (e.g. 0 and 1). If a node is dichotomous (i.e. the two connecting branches have different but in total only 2 states) then we assign a single migration event. In some cases more than one potential

ancestral state for a given node result in an equally minimal number of ancestral changes, and in these cases the ancestral state of this node (and the associated migration events) cannot be unambiguously inferred. We used only unambiguously reconstructed ancestral states and the respective estimated migration events.

Sub-datasets and robustness against sampling bias

To minimize variation in the number of sequences among the different areas, we downsized the number of sequences of countries that have a much larger number of sequences compared to other countries (Dataset I, Table S1). To examine robustness of results to our subsampling we performed a sensitivity analysis by forming two datasets, one larger (Dataset I, Table S1) and one smaller (Dataset II, Table S1). For each of these datasets the sequences were selected randomly from the pool of all available sequences (Initial Dataset, Table S1). The analyses from the two datasets gave very similar statistically significant pathways. We also compared the mean migration events across all pathways inferred from the two datasets using the Spearmans' rank correlation, which confirmed that phylogeographic inference between these two datasets was highly correlated (Spearman rank correlation coefficient $r_s = 0.931$, $p < 0.001$). We present only the results of the larger Dataset I.

Inference of viral migration routes - preliminary analysis

Migration events were estimated from each bootstrap tree having the HIV-1 sequences grouped in different areas according to strategies (1) - (4) (described in the main text). The mean of migration events for each migration pathway estimated from all bootstrap trees for the analyses (1) - (4) are reported in Tables 1, 2 and Tables S2, S3, respectively. The maximum number of different localities among which migration events can be inferred in PAUP is set to 32. Given that in our dataset the number of different regions was 37 (North America, Central & South America, the Caribbean, Africa, Asia, Oceania and 31 European countries), we performed a preliminary phylogeographic analysis in order to combine viral strains from different regions into a single group and reduce the number of geographic groups to 32. This analysis showed that specific European countries appeared as the single source of viral migration towards another European country, which acted as a receiver only and not as a source of dispersion to any other country. Since viral migration among these

countries was deterministic (one epidemic was nested within the other), we merged the viral strains from these countries into groups without compromising the robustness of viral migration among other countries. The countries that were merged were Czech Republic and Slovakia; Estonia and Latvia; Italy and Ireland; and Sweden, Norway and Finland, respectively (Table S1). The country mentioned first within these groups acted as the source of viral migration towards the others (e.g. Czech Republic was the source for Slovakia). Subsequently, we used this particular grouping of countries to perform all phylogeographic analyses (1) - (4).

HIV-1 migration between regions: minor findings

HIV-1 migration between regions and individual countries within Europe: qualitative aspects: minor findings

To describe in detail migratory routes between different geographic regions and European countries (analysis 3), we performed a more detailed phylogeographic analysis in which we separated Europe into distinct countries. Significant migration was found as expected from analysis (1) from North America, Central & South America to several European countries (Table S2). We didn't find many significant migrations between the other geographic regions and European countries (with some minor exceptions). This is not surprising given the different type of analysis where Europe has been separated into different countries. Intuitively, this approach has less power to detect viral migration between regions with a small number of sequences (individual countries) and regions with large number of sequences (geographic areas).

HIV-1 migration within Europe: minor findings

We conducted a distinct phylogeographic sub-analysis to reconstruct the patterns of migration within Europe (analysis 4) (Table S3). The significant migratory routes are shown in two different ways: i) in different colors, depicting the direction of migratory pathways for all routes (Figure S3B) and ii) for pathways between Western and Eastern Europe (Figure S5). Additionally we show the significant migratory routes in a chart, plotting for each country the number of locations for which we found significant migration (importing and exporting) (Figure S6). Similarly the bars are located on each country in a map relative to the background of the proportion of subtype B infections as estimated from a recent study in Europe (Figure S3A) (4).

According to Figure 3, significant migration was observed for all countries included in the analysis; however we observed large differences in the numbers of significantly more connected countries across Europe. For Italy, Germany and Spain the number of exporting areas within their network was more than 15 while for Belgium, Portugal, Netherlands, Poland, and Denmark it was between 10-15 (Figure 3). The pattern varies across Europe with respect to significant importing migration. For example the highest incoming connectivity in terms of the number of countries was found for Czech Republic/Slovakia, Austria and Italy (number of countries from which viral spread was detected: 12-15). High importing network was found also for

Germany, Luxembourg (Germany, Spain, Belgium, Portugal, Netherlands and Poland (number of importing countries: 9-11). For Czech Republic/Slovakia, Luxembourg and Austria showing the highest network of incoming migration, the opposite pattern was found for the exporting network. In contrast, for Italy, Germany, Spain, Belgium, Portugal, Netherlands and Poland we detected bidirectional migration. We detected the highest complexity for Italy, Germany and Spain, suggesting that these countries are major sources for the spread of HIV-1 subtype B infection across Europe, which is also compatible with them having high numbers of subtype B infections (>40% of the European). We note however that the latter areas should not be considered as the only sources of viral dissemination within Europe but rather as locations with the highest connectivity "sources" for viral migration across Europe. The Figure S7 illustrates migration pathways within Europe with the highest ratios of observed over expected mean migration events.

Study characteristics and limitations

We have studied the global patterns of subtype B migration on the largest systematically collected sample. Can our study of the global pattern for the subtype B epidemic be reliable even though we used a small sample of sequences across the globe? Can it be representative? Given that the HIV sequences were not part of a study fulfilling certain criteria for representativeness, representativeness might not hold in some cases. We believe that our study is robust for several reasons. Firstly because HIV sequences were systematically collected, either through meta-analyses approach or within prospective studies. Secondly, given that subtype B is prevalent in most developed countries, the exclusion of small geographic regions or those where subtype B is not prevalent is unlikely to compromise the validity of our findings. Thirdly, we have assessed sampling bias by analyzing datasets constructed with different sampling strategies and our results are consistent. Fourth, we have successfully recovered previously described monophyletic (locally dispersed) epidemics from Asia and Caribbean (5-9). Finally, and most importantly, our findings are epidemiologically coherent; the exporting viral migration estimates correlate with the prevalent cases of subtype B per country suggesting a mechanistic link between the "load" of the country and the probability of spillover to another country.

References

1. **Los Alamos National Laboratory** 2005-2006, posting date. Los Alamos National Laboratory, HIV Databases. [Online.]
2. **Struck D, Lawyer G, Ternes AM, Schmit JC, Bercoff DP.** 2015. COMET: adaptive context-based modeling for ultrafast HIV-1 subtype identification. Nucleic acids research **42:**e144.
3. **Paraskevis D, Magiorkinis E, Magiorkinis G, Sypsa V, Paparizos V, Lazanas M, Gargalianos P, Antoniadou A, Panos G, Chrysos G, Sambatakou H, Karafoulidou A, Skoutelis A, Kordossis T, Koratzanis G, Theodoridou M, Daikos GL, Nikolopoulos G, Pybus OG, Hatzakis A.** 2007. Increasing prevalence of HIV-1 subtype a in Greece: Estimating epidemic history and origin. Journal of Infectious Diseases **196:**1167-1176.
4. **Abecasis AB, Wensing AM, Paraskevis D, Vercauteren J, Theys K, Van de Vijver DA, Albert J, Asjo B, Balotta C, Beshkov D, Camacho RJ, Clotet B, De Gascun C, Griskevicius A, Grossman Z, Hamouda O, Horban A, Kolupajeva T, Korn K, Kostrikis LG, Kucherer C, Liitsola K, Linka M, Nielsen C, Otelea D, Paredes R, Poljak M, Puchhammer-Stockl E, Schmit JC, Sonnerborg A, Stanekova D, Stanojevic M, Struck D, Boucher CA, Vandamme AM.** 2013. HIV-1 subtype distribution and its demographic determinants in newly diagnosed patients in Europe suggest highly compartmentalized epidemics. Retrovirology **10:**7.
5. **Cho YK, Jung YS, Foley BT.** 2011. Phylogenetic analysis of full-length pol gene from Korean hemophiliacs and plasma donors infected with Korean subclade B of HIV type 1. AIDS Res Hum Retroviruses **27:**613-621.
6. **Cleghorn FR, Jack N, Carr JK, Edwards J, Mahabir B, Sill A, McDanal CB, Connolly SM, Goodman D, Bennetts RQ, O'Brien TR, Weinhold KJ, Bartholomew C, Blattner WA, Greenberg ML.** 2000. A distinctive clade B HIV type 1 is heterosexually transmitted in Trinidad and Tobago. Proceedings of the National Academy of Sciences of the United States of America **97:**10532-10537.
7. **Tsui SK, Fong NY, Li SK, Leung KK, Chan DP, Chan PK, Wong KH, Lee SS.** 2010. Full genome analysis of an emerging cluster of human immunodeficiency virus type 1 subtype B infection in Hong Kong. AIDS Res Hum Retroviruses **26:**117-122.
8. **Vaughan HE, Cane P, Pillay D, Tedder RS.** 2003. Characterization of HIV type 1 clades in the Caribbean using pol gene sequences. AIDS Res Hum Retroviruses **19:**929-932.

9. **Wang W, Jiang S, Li S, Yang K, Ma L, Zhang F, Zhang X, Shao Y.** 2008. Identification of subtype B, multiple circulating recombinant forms and unique recombinants of HIV type 1 in an MSM cohort in China. AIDS Res Hum Retroviruses **24:**1245-1254.

Supplemental Tables

Table S1. Number of sequences per country. The Initial Dataset describes the number of sequences per country downloaded after the bibliographic search. Datasets I and II were used in the analyses.

Table S2. A: Means of observed migration events across all bootstrap trees between large geographic regions and European countries. B: Ratio of mean of observed over mean of expected migration events between large geographic regions and European countries

Table S3. A: Means of observed migration events across all bootstrap trees between the European countries. B: Ratio of mean of observed over mean of expected migration events between the European countries

Table S4. List of studies fulfilling selection criteria.

Supplemental Figures

Figure S1. Global migration patterns of HIV-1 subtype B estimated by statistical phylogeography under the geographical grouping strategy 2. Colors indicate different geographic regions (highlighted countries) from which HIV-1 sequences were available. Arrows indicate the direction of subtype B spread. The thickness of the arrows is proportional to the ratio of observed/expected migration events (mean values) between different regions (Table 2). Dots for different geographic areas are placed in the center of each area.

Figure S2. ML phylogeographic tree showing viral clades in different colors according to Western European, Central/Eastern European and non-European sampling.

Figure S3. A: Number of exporting (green bar) and importing (red bar) pathways for each country/region in Europe. The background blue color tone in each country/region is analogous to the proportion of HIV-1 epidemic due to subtype B with darker color indicating a higher proportion of subtype B infections. B: Intra-European significant migration pathways of HIV-1 subtype B estimated by statistical phylogeography. Orange lines indicate bidirectional viral migration while yellow-black lines show the direction of viral migration from the source (yellow) to the target (black). Background blue colors are as in A.

Figure S4. Ratio of observed versus expected out-of-Europe migration index for migration events between geographic areas and specific Western European countries. Country names are shown in ISO three-letter codes and are as in Figure 1. Only Western European countries with significant migration events with at least one non-European geographic area are reported.

Figure S5. Intra-European migration patterns of HIV-1 subtype B estimated by statistical phylogeography. Lines correspond to migration pathways between Western and Central/Eastern European countries.

Figure S6. Number of significant exporting and importing migration pathways per country/region. Only intra-European migration is shown. Countries from Central/Eastern Europe are in green circles. Country names are shown in ISO three-letter codes and are as in Figure 1.

Figure S7. Migration pathways with the highest ratios of observed/expected mean events (Table S2). The thickness of the lines is proportional to the ratio of observed/expected migration events across the pathways.
