# Supplementary material for: The global spread of HIV-1 subtype B epidemic
Source: Infect Genet Evol. 2016 Dec;46:169–79. doi: 10.1016/j.meegid.2016.05.041 (PMC5157885; doi:10.1016/j.meegid.2016.05.041)
Supplement: Supplementary file 2 — Supplementary figures. [file mmc2.pdf]

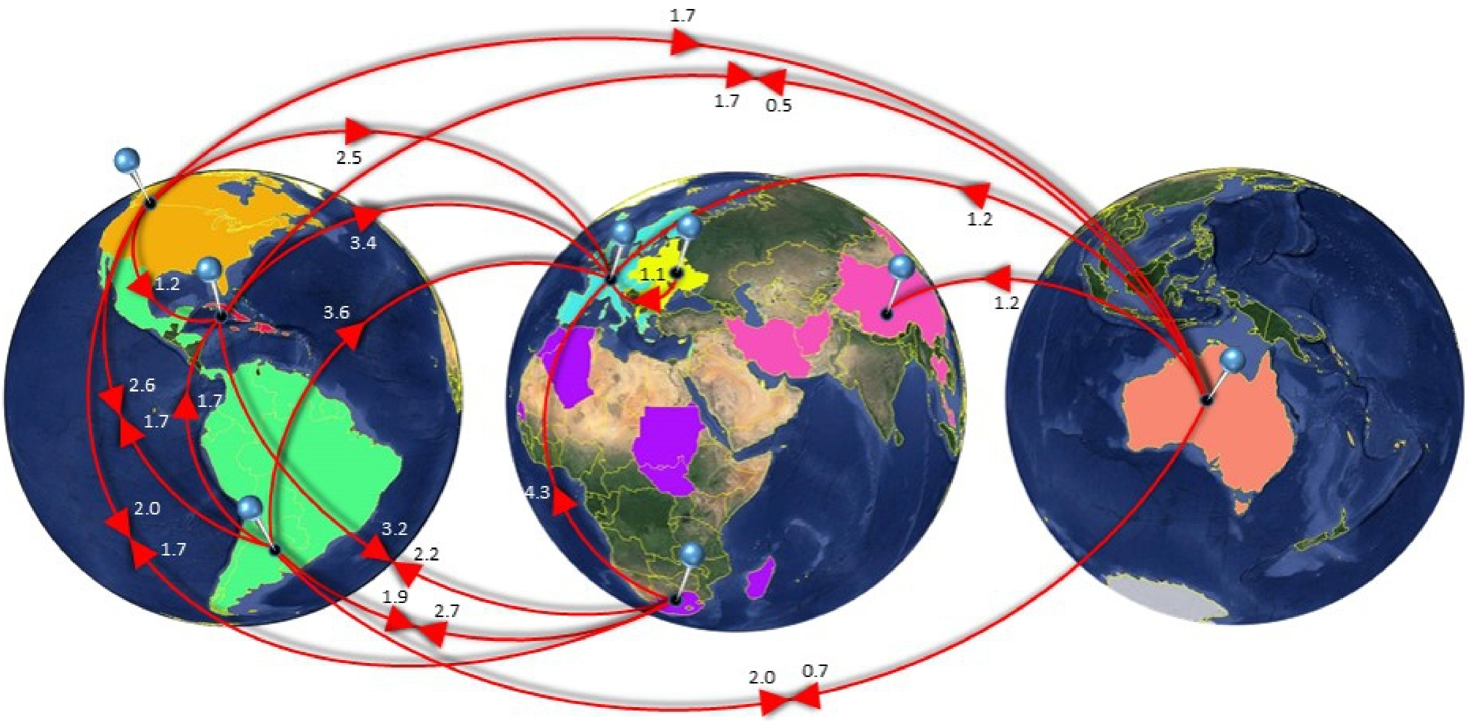

Figure S1. Global migration patterns of HIV-1 subtype B estimated by statistical phylogeography under the geographical grouping strategy 2. Colors indicate different geographic regions (highlighted countries) from which HIV-1 sequences were available. Pins represent different geographic regions (group of highlighted countries). Arrows indicate the direction of subtype B spread and the number next to each arrow represents the ratio of mean of observed over mean of expected events.

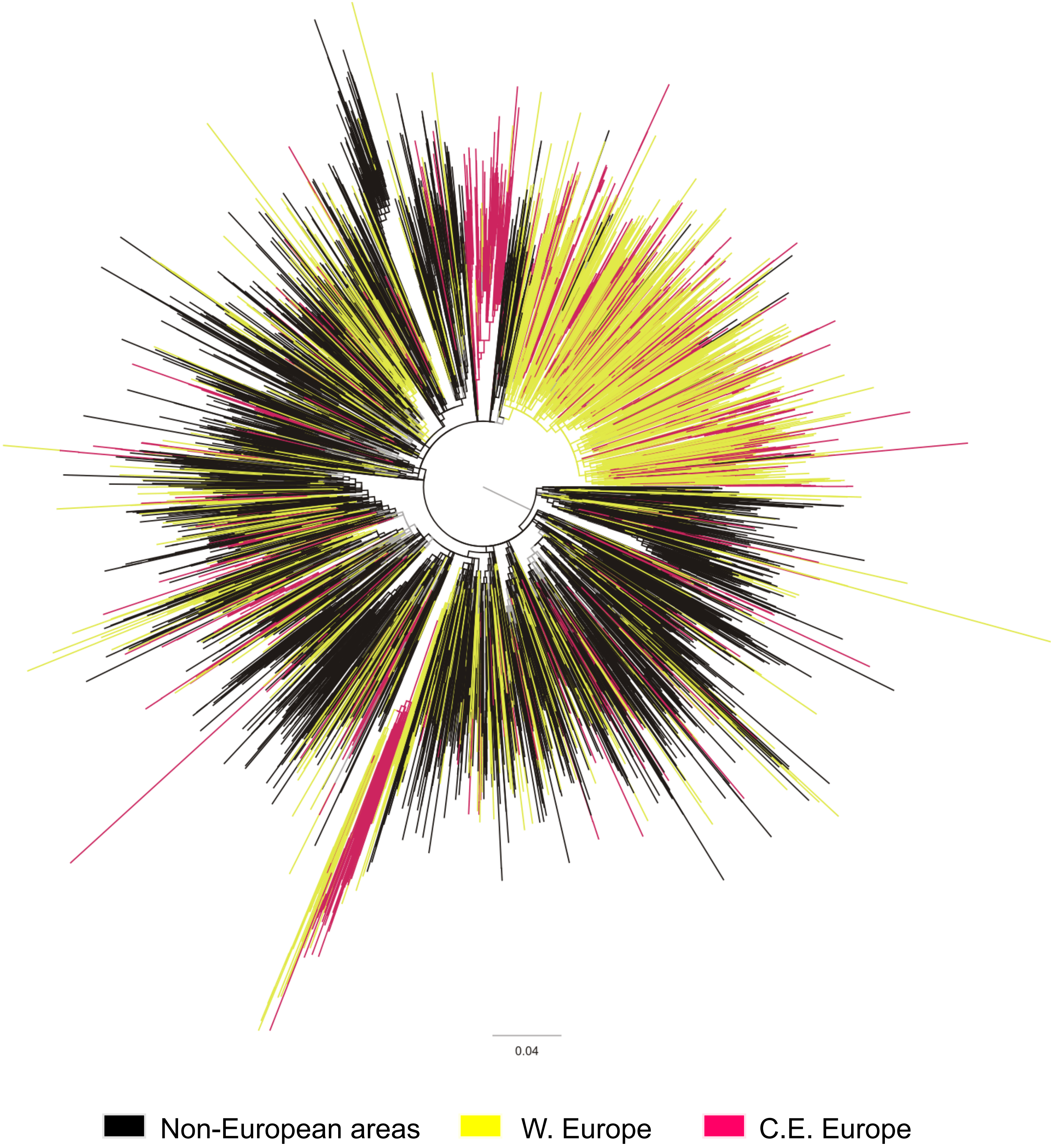

Figure S2. ML phylogeographic tree showing viral clades in different colors according to Western European, Central/Eastern European and non-European sampling. The rooting of the tree is midpoint.

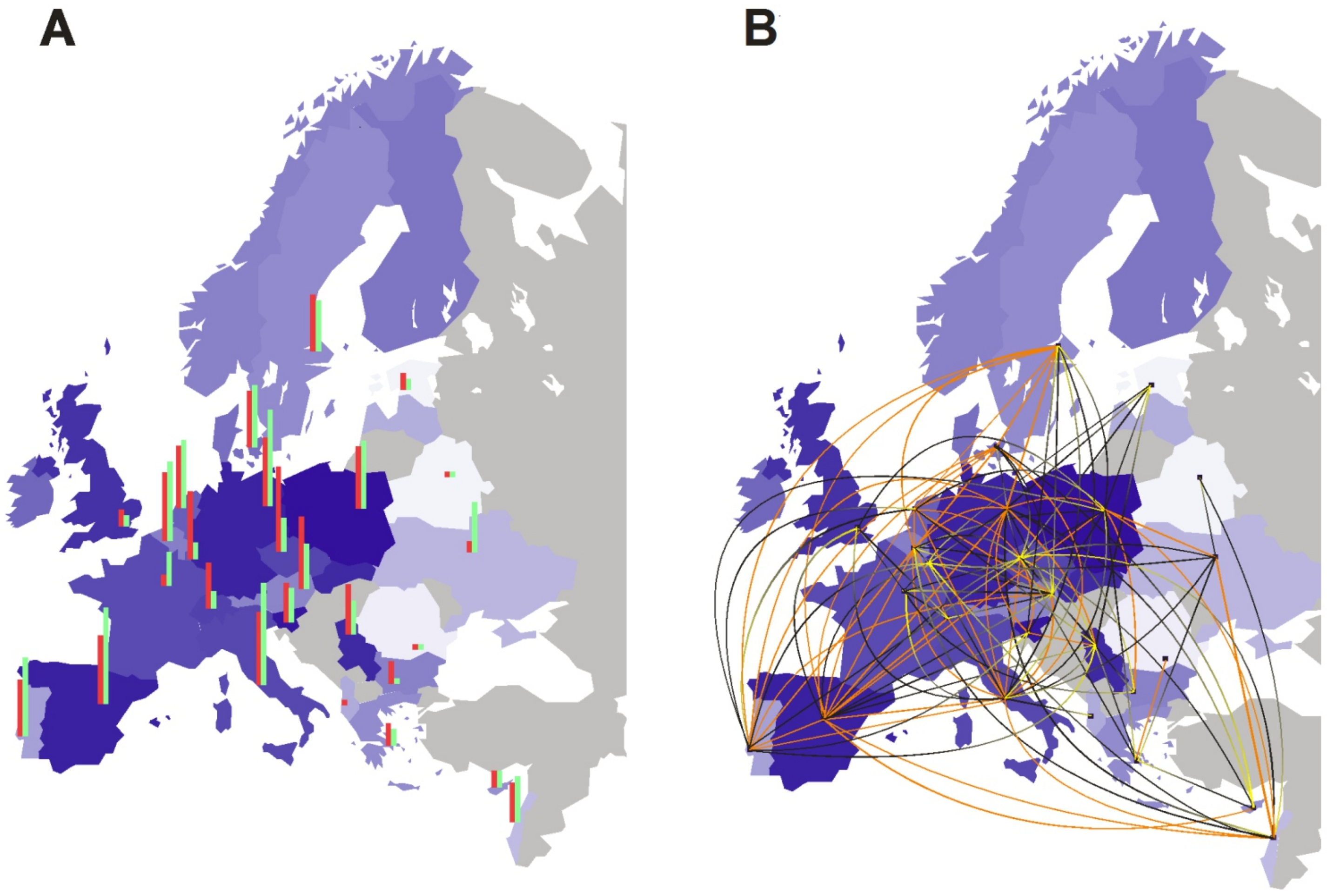

Figure S3. A: Number of exporting (green bar) and importing (red bar) pathways for each country/region in Europe. The background blue color tone in each country/region is analogous to the proportion of HIV-1 epidemic due to subtype B with darker color indicating higher percentage of subtype B infections. B: Intra-European significant dispersal pathways of HIV-1 subtype B estimated by statistical phylogeography. Orange lines indicate bidirectional viral dispersal while yellow-black lines show the direction of viral migration from the source (yellow) to the target (black). Background blue colors are as in A.

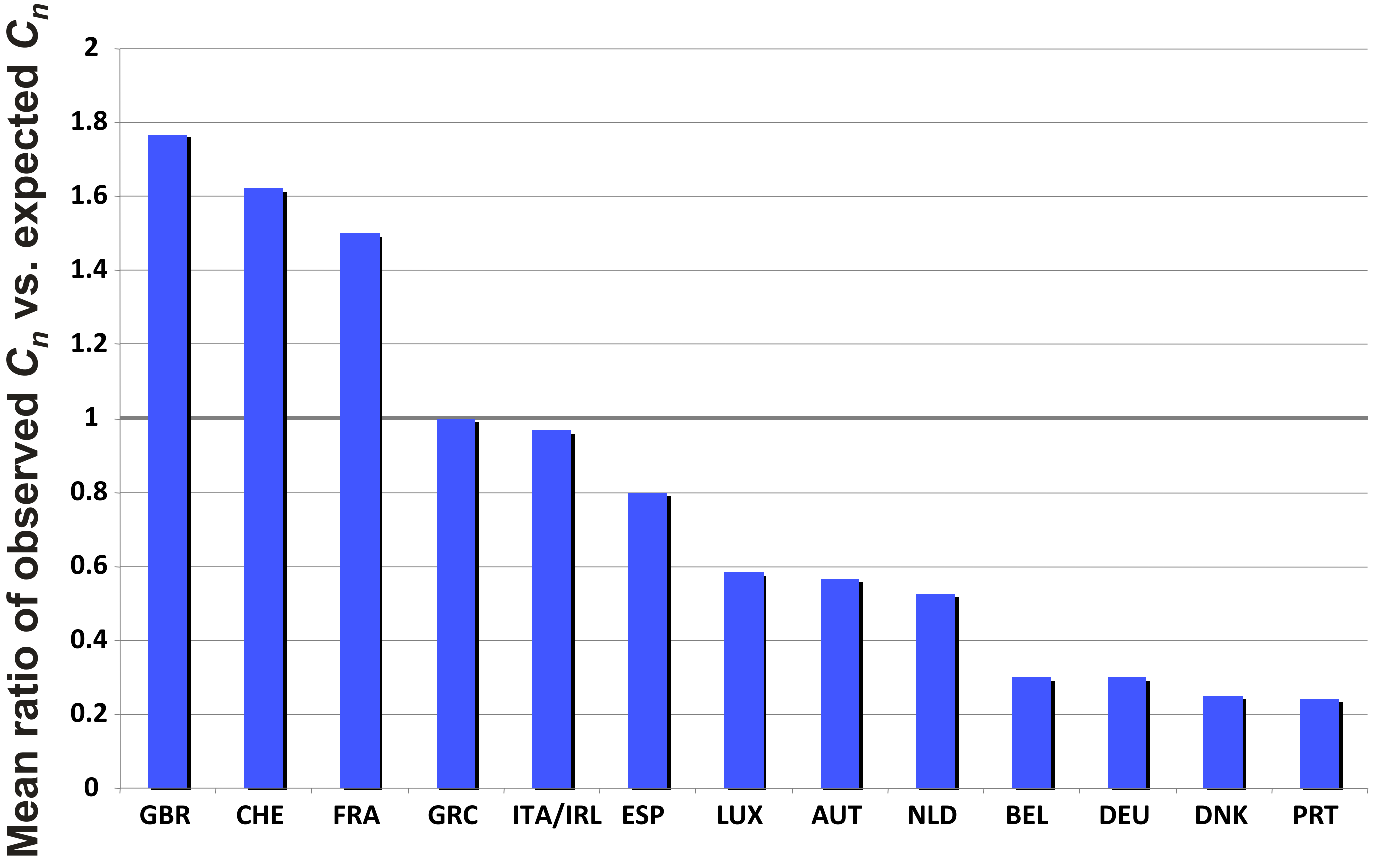

Figure S4. Ratio of observed versus expected out-of-Europe migration index for migration events between geographic areas and specific Western European countries. Country names are shown in ISO three-letter codes and are as in Figure 4. Only Western European countries with significant migration events with at least one non-European geographic area are reported.

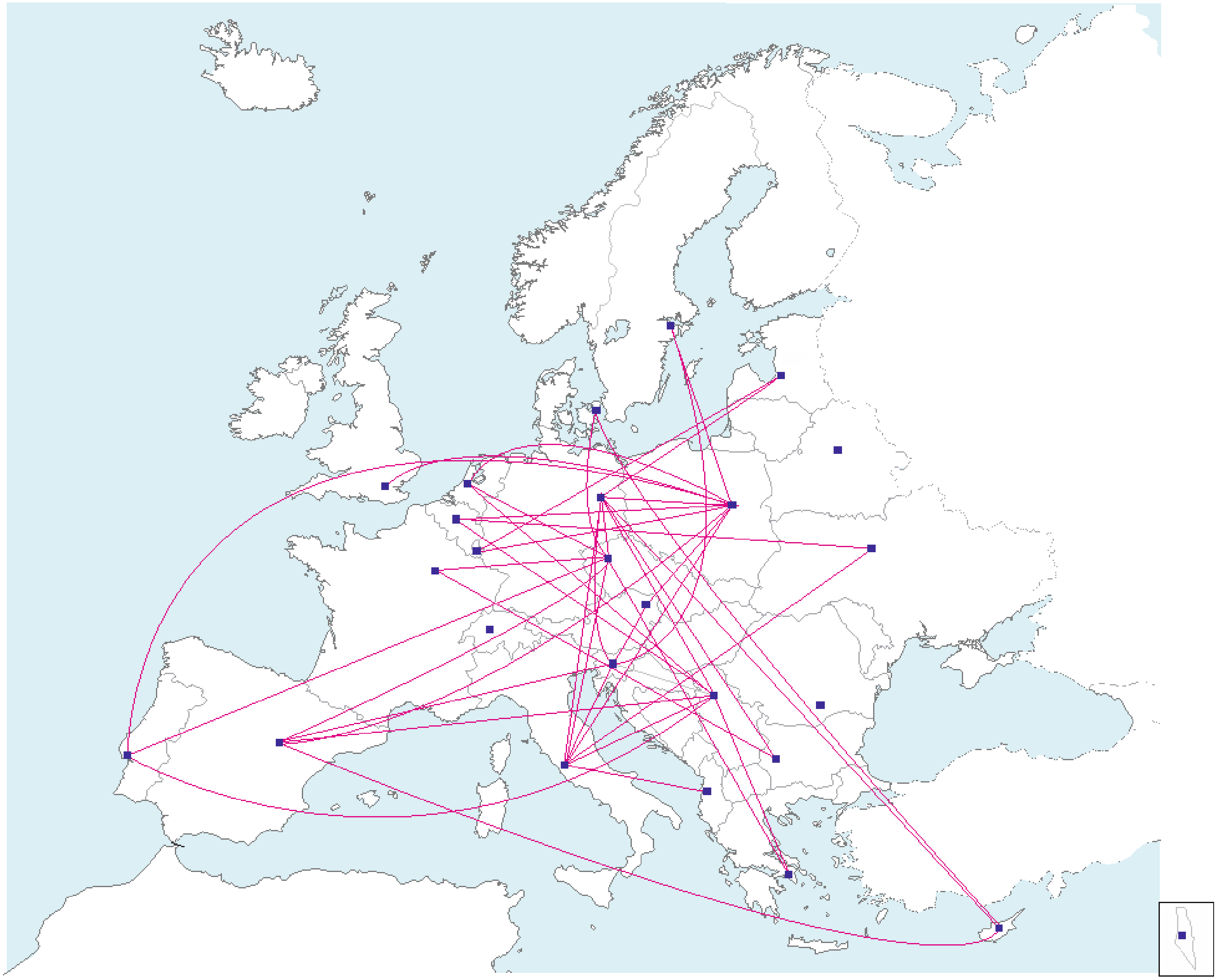

Figure S5. Intra-European dispersal patterns of HIV1- subtype B estimated by statistical phylogeography. Llines correspond to dispersal pathways between Western and Central/Eastern European countries.

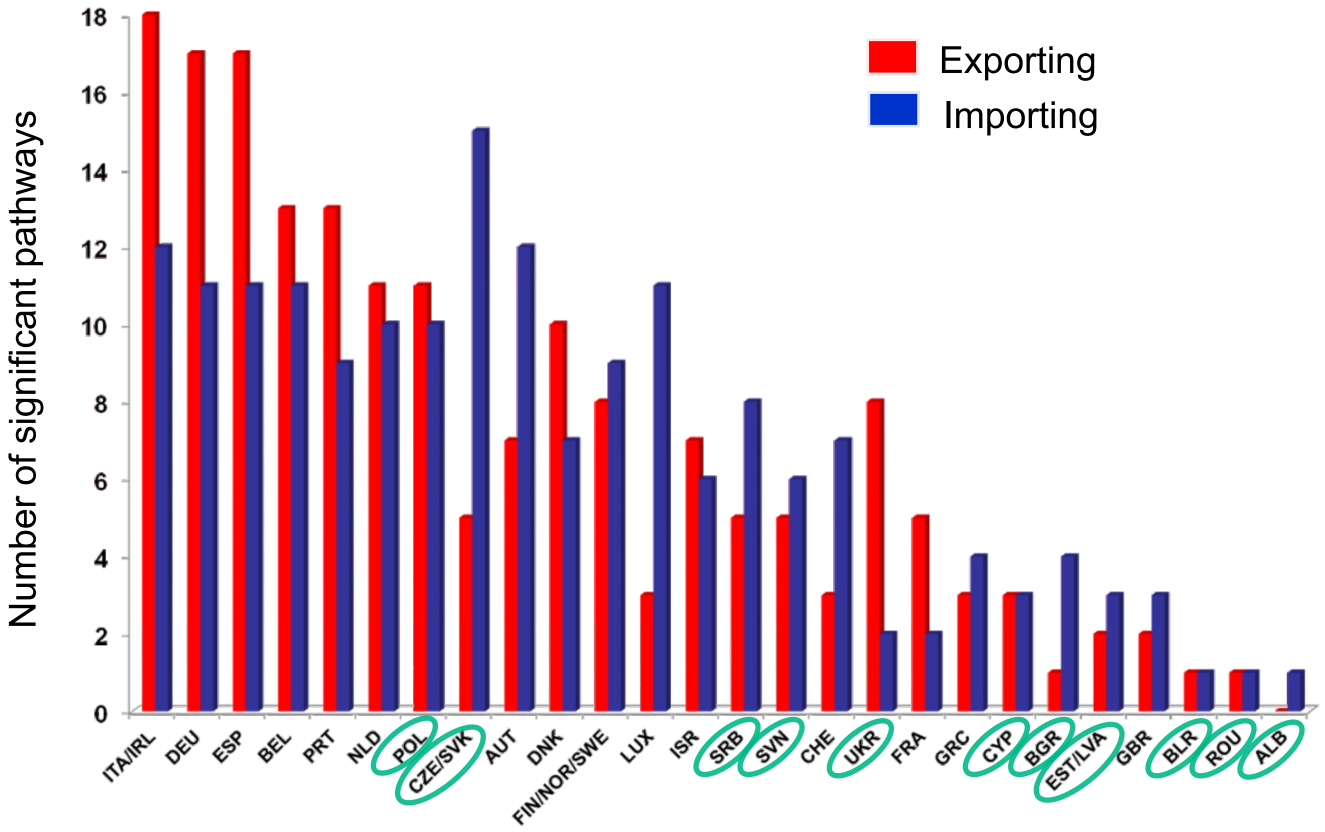

Figure S6. Number of significant exporting and importing migration pathways per country/region. Only intra-European dispersal is shown. Countries from Central/Eastern Europe are in green circles. Country names are shown in ISO three-letter codes and are as in Figure 1.

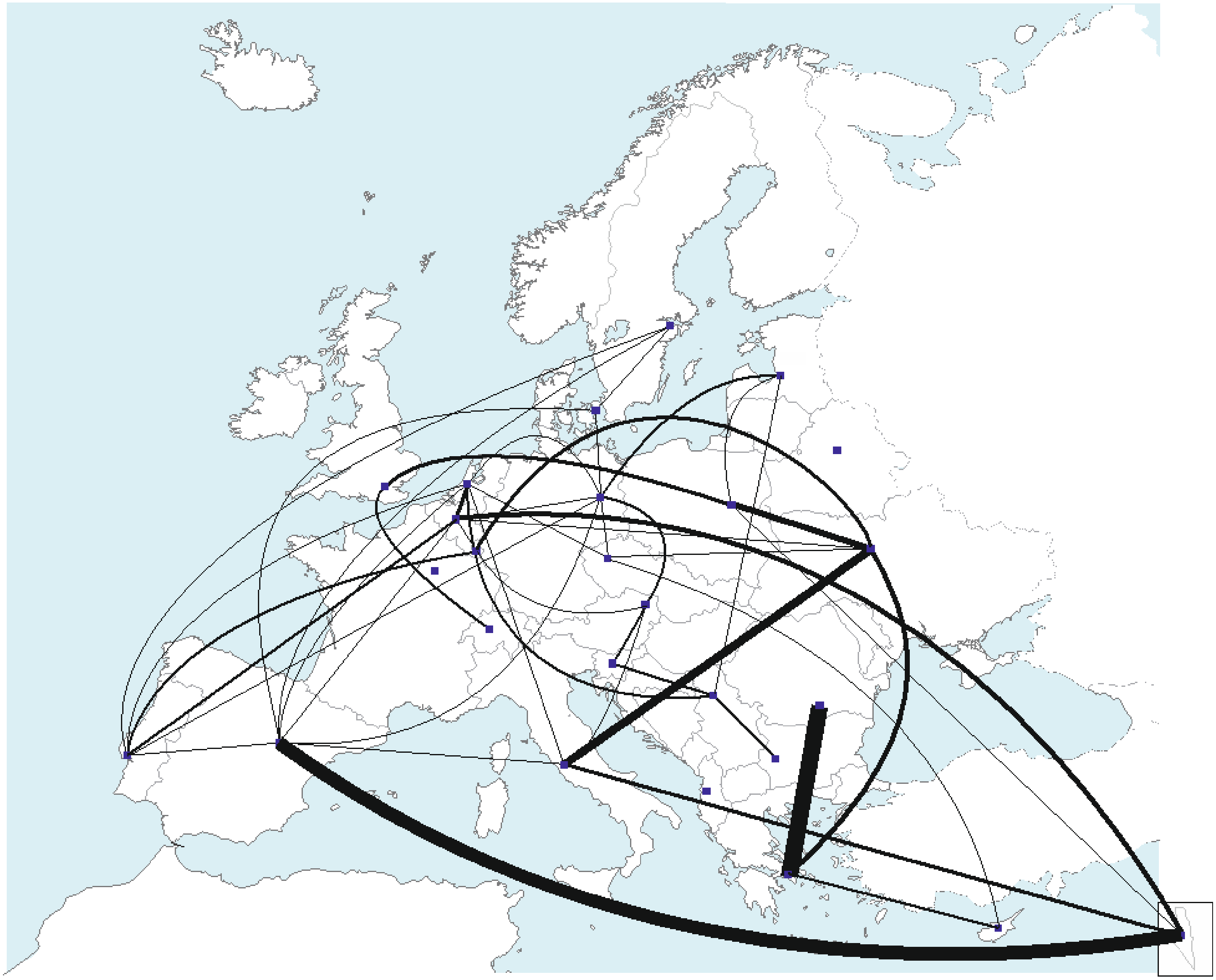

Figure S7. Dispersal pathways with the highest ratios of observed/expected mean events (Table S2). The thickness of the lines is proportional to the ratio of observed/expected migration events across the pathways.
