# Supplementary material for: The global spread of HIV-1 subtype B epidemic
Source: Infect Genet Evol. 2016 Dec;46:169–79. doi: 10.1016/j.meegid.2016.05.041 (PMC5157885; doi:10.1016/j.meegid.2016.05.041)
Supplement: Supplemental Table 1 — Number of sequences per country. The Initial Dataset describes the number of sequences per country downloaded after the bibliographic search. Datasets I and II were used in the analyses. [file mmc3.pdf]

Supplemental Table 1. Number of sequences per country. The Initial Dataset describes the number of sequences per country downloaded after the bibliographic search. Datasets I and II were used in the analyses.

Number of subtype B sequences per country in the Datasets

Sampling Region	Initial Dataset	Dataset I	Dataset II	
**North America**				
Canada (CAN)	870	250	86	
San Francisco (USA)	33	33	22	
Denver (USA)	38	38	22	
Boston (USA)	96	96	22	
New York (USA)	123	123	22	
Seattle (USA)	83	83	22	
Rest regions (USA)	1295	1295	54	
**Total North America**	**2538**	**1918**	**250**	
**Central & South America**				
Mexico (MEX)	9	9	9	
El Salvador (SLV)	8	8	8	
Honduras (HND)	4	4	4	
Beliz (BLZ)	3	3	3	
Panama (PAN)	13	13	13	
Brazil (BRA)	438	250	36	
Argentina (ARG)	164	164	36	
Chile (CHL)	115	115	36	
Colombia (COL)	35	35	35	
Guyana (GUY)	6	6	6	
Suriname (SUR)	5	5	5	
Ecuador (ECU)	2	2	2	
Bolivia (BOL)	1	1	1	
Peru (PER)	11	11	11	
Paraguay (PRY)	3	3	3	
Uruguay (URY)	6	6	6	
Venezuela (VEN)	239	239	36	
**Total Central & South America**	**1062**	**874**	**250**	
**Carribean**				
Antigua and Barbuda (ATG)	9	9	9	
Bahamas (BHS)	11	11	11	
Cuba (CUB)	221	221	91	
Puerto Rico (PRI)	5	5	5	
Dominica (DMA)	3	3	3	
Dominican Republic (DOM)	22	22	22	
Grenada (GRD)	5	5	5	
Haiti (HTI)	23	23	23	
Montserrat (MSR)	1	1	1	
Saint Lucia (LCA)	4	4	4	
Saint Vincent (VCT)	6	6	6	
Jamaica (JAM)	6	6	6	
Trinidad and Tobago (TTO)	64	64	64	
**Total Carribean**	**380**	**380**	**250**	
**Africa**				
Algeria (DZA)	68	68	68	
Madagascar (MDG)	5	5	5	
Morocco (MAR)	56	56	56	
Senegal (SEN)	13	13	13	
Seychelles (SYC)	9	9	9	
South Africa (ZAF)	4	4	4	
Sudan (SDN)	1	1	1	
**Total Africa**	**156**	**156**	**156**	
**Oceania**				
Australia (AUS)	208	208	208	
**Asia**				
China (CHN)	106	106	61	
Hong-Kong (HKG)	3	3	3	
Iran (IRN)	12	12	12	
Japan (JPN)	371	371	62	
South Korea (PRK)	39	39	39	
Taivan (TWN)	271	271	61	
Thailand (THA)	12	12	12	
**Total Asia**	**814**	**814**	**250**	
**Europe**				
Albania (ALB)	22	22	22	
Austria (AUT)	117	117	117	
Belgium (BEL)	221	221	221	
Bulgary (BGR)	90	90	90	
Belarus (BLR)	6	6	6	
Switzerland (CHE)	160	160	160	
Serbia & Montenegro(SRB)	115	115	115	
Cyprus (CYP)	107	107	107	
Czech Republic & Slovakia (CZE)/(SVK)	344	344	250	
Germany (DEU)	877	249	250	
Denmark (DNK)	226	226	226	
Esthoia and Latvia (EST)/(LVA)	22	22	22	
Spain (ESP)	344	344	250	
France (FRA)	279	279	250	
Greece (GRC)	132	132	132	
Israel (ISR)	35	35	35	
Italy & Ireland (ITA)/(IRL)	528	256	250	
Luxembourg (LUX)	123	123	123	
Netherlands (NLD)	83	83	83	
Finland, Norway & Sweden (FIN)/(NOR)/(SWE)	327	327	250	
Poland (POL)	251	251	250	
Portugal (PRT)	146	146	146	
Slovenia (SVN)	153	153	153	
Ukraine (UKR)	50	50	50	
United Kingdom (GBR)	91	91	91	
Romania (ROU)	71	71	71	
**Total Europe**	**4920**	**4020**	**3720**	
**Total**	**10078**	**8370**	**5084**	

Note.– Countries' codes are according to ISO.
