# Supplementary material for: The global spread of HIV-1 subtype B epidemic
Source: Infect Genet Evol. 2016 Dec;46:169–79. doi: 10.1016/j.meegid.2016.05.041 (PMC5157885; doi:10.1016/j.meegid.2016.05.041)
Supplement: Supplemental Table 2 — A. Means of observed migration events across all bootstrap trees between large geographic regions and European countries. B. Ratio of mean of observed over mean of expected migration events between large geographic regions and European countries. [file mmc4.pdf]

Supplemental Table 2

A. Means of observed migration events across all bootstrap trees between large geographic regions and European countries

From \ To	N.America	C.S.America	Caribbean	Africa	Asia	Oceania	ALB	AUT	BEL	BGR	BLR	CHE	SRB	CYP	CZE/SVK	DEU	DNK	EST/LVA	ESP	FRA	GRC	ISR	ITA/IRL	LUX	NLD	FIN/NOR/SWE	POL	PRT	SVN	UKR	GBR	ROU
**N.America**		**11.172**	**5.984**	**5.092**	**4.860**	**5.904**	0.552	**2.576**	**3.760**	1.364	0.080	**7.376**	1.764	**3.368**	3.112	1.748	**5.804**	0.476	**4.440**	**9.456**	2.400	**0.940**	**6.492**	**4.104**	1.240	**4.244**	0.860	1.264	1.196	0.348	**5.304**	0.012
**C.S.America**	**10.124**		**6.572**	**4.968**	**5.008**	**5.388**	**0.880**	1.988	2.688	1.484	0.108	**6.472**	1.444	**3.384**	3.064	1.560	3.524	0.396	4.072	**8.180**	2.408	**1.076**	**6.508**	**3.848**	1.356	3.704	0.624	2.168	0.996	0.316	**4.052**	0.012
**Caribbean**	1.468	2.804		2.284	1.172	1.776	0.064	0.656	0.400	0.152	0.008	0.900	0.224	0.712	0.352	0.224	0.676	0.112	0.492	1.132	0.820	0.112	2.128	0.592	0.400	0.420	0.068	0.192	0.264	0.064	0.996	0.000
**Africa**	1.068	1.272	0.844		0.592	0.688	0.068	0.624	0.340	0.200	0.032	0.820	0.316	0.452	0.340	0.196	0.456	0.016	0.692	**1.588**	0.332	0.320	**1.520**	0.560	0.152	0.436	0.048	0.172	0.184	0.032	**0.712**	0.000
**Asia**	0.720	1.628	1.184	1.076		1.772	0.056	0.180	0.260	0.112	0.012	0.968	0.132	0.200	0.268	1.152	0.304	0.040	0.236	0.656	0.356	0.072	0.376	0.268	0.084	1.000	0.040	0.064	0.108	0.016	0.304	0.000
**Oceania**	1.388	1.664	1.680	0.624	2.224		0.068	0.528	0.520	0.144	0.032	1.044	0.204	0.716	1.064	0.764	0.844	0.064	0.596	1.272	0.404	0.096	1.672	0.520	0.424	0.752	0.332	0.148	0.232	0.028	0.936	0.004
**ALB**	0.004	0.004	0.000	0.012	0.008	0.000		0.000	0.004	0.004	0.000	0.044	0.004	0.004	0.004	0.008	0.000	0.000	0.000	0.004	0.056	0.000	0.044	0.000	0.016	0.000	0.000	0.000	0.012	0.000	0.012	0.000
**AUT**	0.220	0.208	0.132	0.280	0.060	0.188	0.032		0.288	0.048	0.000	0.312	0.156	0.088	0.488	0.240	0.260	0.004	0.204	0.424	0.024	0.052	0.428	0.340	0.244	0.480	0.132	0.172	0.124	0.012	0.184	0.000
**BEL**	0.572	0.644	0.296	0.260	0.180	0.528	0.036	1.116		0.104	0.088	1.020	0.556	0.332	1.860	**4.648**	2.344	0.016	**3.276**	0.520	0.080	0.188	2.320	**3.312**	**3.988**	2.388	1.044	**2.244**	0.744	0.032	0.348	0.000
**BGR**	0.032	0.044	0.024	0.028	0.032	0.012	0.000	0.016	0.048		0.020	0.048	0.024	0.028	0.016	0.028	0.040	0.004	0.028	0.068	0.008	0.008	0.028	0.016	0.004	0.036	0.144	0.016	0.008	0.028	0.036	0.008
**BLR**	0.000	0.000	0.000	0.000	0.004	0.000	0.000	0.000	0.000	0.000		0.000	0.000	0.000	0.000	0.000	0.000	0.000	0.000	0.004	0.000	**0.268**	0.000	0.000	0.000	0.000	0.000	0.000	0.000	0.000	0.000	0.000
**CHE**	**1.652**	**1.660**	0.932	**1.240**	0.696	0.984	**0.272**	0.624	1.188	0.332	**0.120**		0.784	0.564	0.532	0.528	1.008	0.176	1.264	**1.656**	0.532	0.240	**1.824**	0.744	0.444	0.716	0.172	0.412	0.312	0.056	**0.768**	0.000
**SRB**	0.112	0.172	0.132	0.172	0.080	0.068	0.000	0.208	0.112	**0.624**	0.000	0.284		0.080	0.196	**1.372**	0.148	0.120	0.260	0.208	0.096	0.028	0.172	0.084	0.052	0.168	0.032	0.172	**0.844**	0.004	0.080	0.000
**CYP**	0.300	0.252	0.200	0.352	0.120	0.212	0.040	0.108	0.124	0.136	0.004	0.300	0.076		**1.204**	0.132	0.164	0.024	**0.680**	0.344	**1.140**	0.024	0.172	0.196	0.084	0.172	0.036	0.056	0.044	0.020	0.200	0.000
**CZE/SVK**	0.312	0.392	0.164	0.132	0.108	0.308	0.024	0.756	0.668	0.104	0.004	0.236	0.148	0.188		1.152	0.488	0.024	0.888	0.396	0.088	0.104	0.656	0.256	0.388	0.672	0.308	0.396	0.448	0.012	0.128	0.000
**DEU**	0.644	0.680	0.348	0.232	0.200	0.892	0.032	**5.668**	**9.336**	1.000	0.012	1.456	**2.596**	1.516	**6.620**		**7.332**	**1.108**	**9.888**	1.928	0.140	0.676	**6.552**	1.776	**4.612**	**7.048**	**5.088**	**5.672**	2.068	0.088	1.048	0.004
**DNK**	0.908	1.024	0.584	0.448	0.368	0.640	0.044	0.856	**2.608**	0.144	0.004	0.816	0.572	1.476	1.172	1.808		0.020	1.836	1.056	0.208	0.220	1.888	0.540	0.956	**7.584**	0.640	1.040	0.448	0.024	0.528	0.012
**EST/LVA**	0.072	0.040	0.052	0.012	0.052	0.028	0.008	0.004	0.016	0.004	0.000	0.064	0.000	0.040	0.024	0.020	0.000		0.032	0.052	0.008	0.000	0.004	**0.340**	0.000	0.008	0.000	0.004	0.008	0.088	0.064	0.000
**ESP**	1.052	1.528	0.660	0.704	0.316	0.648	0.060	**2.660**	**7.740**	0.144	0.004	1.548	2.172	1.040	**5.064**	**8.824**	**5.552**	0.020		1.156	0.264	0.712	**6.460**	1.628	**3.720**	**7.656**	3.520	**6.540**	1.520	0.028	0.732	0.180
**FRA**	**7.536**	**7.964**	2.408	**3.784**	2.740	**3.664**	0.252	1.596	1.888	**2.064**	0.100	**3.864**	0.984	1.764	**4.080**	**0.952**	2.172	0.276	2.456		1.120	0.564	**3.964**	**2.284**	0.720	2.216	0.572	0.944	1.344	0.044	**2.672**	0.000
**GRC**	0.148	0.676	0.692	0.372	0.072	0.104	0.064	0.048	0.024	0.004	0.000	0.176	0.392	0.348	0.600	0.032	0.072	0.008	0.060	0.132		0.028	0.088	0.036	0.024	0.064	0.012	0.008	0.020	0.004	0.068	**2.884**
**ISR**	0.020	0.040	0.020	0.032	0.000	0.028	0.008	0.008	**0.636**	0.004	0.008	0.028	0.000	0.008	**0.748**	0.016	0.012	0.000	**0.416**	0.028	0.016		0.020	**0.320**	0.012	0.020	0.088	0.020	0.000	**0.136**	0.008	0.000
**ITA/IRL**	2.684	**4.548**	1.804	**6.412**	0.836	1.140	**0.760**	**6.348**	**3.860**	0.760	0.012	**4.760**	**2.184**	1.396	1.840	3.456	**4.520**	0.060	**3.820**	**6.268**	0.460	**0.848**		**2.644**	**2.064**	2.552	1.296	**2.808**	2.532	0.048	1.548	0.000
**LUX**	0.356	0.316	0.208	0.264	0.140	0.284	0.044	0.200	0.304	0.036	0.008	0.436	0.104	0.116	0.276	0.144	0.212	0.020	0.184	0.472	0.072	0.032	0.392		0.420	0.232	0.076	**0.976**	0.092	0.012	0.220	0.000
**NLD**	0.052	0.128	0.056	0.052	0.024	0.092	0.008	0.208	**1.544**	0.004	0.000	0.084	0.100	0.100	0.240	0.448	**0.652**	0.000	0.412	0.040	0.008	0.032	0.296	0.256		0.364	0.184	**0.392**	0.036	0.004	0.056	0.000
**FIN/NOR/SWE**	0.700	0.760	0.408	0.404	0.376	0.472	0.032	0.556	2.636	0.124	0.004	0.540	0.308	0.432	1.140	1.956	**3.716**	0.020	2.048	0.488	0.168	0.244	1.420	0.416	0.904		0.688	1.284	0.404	0.028	0.376	0.008
**POL**	0.064	0.108	0.044	0.020	0.020	0.048	0.000	0.612	0.596	1.212	0.040	0.132	0.100	0.048	1.360	2.624	0.356	**0.776**	1.644	0.120	1.392	0.600	0.336	0.544	0.264	2.180		0.376	0.068	0.808	0.040	0.000
**PRT**	0.220	0.232	0.076	0.092	0.068	0.112	0.012	**0.812**	**2.440**	0.040	0.008	0.332	0.484	0.212	0.792	**2.464**	**1.652**	0.000	**2.008**	0.228	0.048	0.216	**1.372**	**2.428**	**1.448**	**2.428**	0.712		0.280	0.000	0.136	0.000
**SVN**	0.068	0.060	0.044	0.052	0.032	0.060	0.000	**1.896**	0.144	0.084	0.000	0.080	**1.948**	0.068	0.660	0.636	0.120	0.000	0.068	0.084	0.000	0.012	0.096	0.056	0.032	**1.028**	0.028	0.040		0.000	0.048	0.000
**UKR**	0.048	0.096	**0.660**	0.020	0.008	0.012	0.004	0.008	**0.260**	**0.108**	**1.416**	0.120	0.004	**0.136**	**0.256**	0.172	0.044	**0.088**	0.160	0.032	**0.184**	**1.740**	0.024	**0.220**	0.000	**0.184**	**0.788**	0.008	0.000		0.012	0.000
**GBR**	**0.668**	**0.720**	0.416	**0.460**	0.236	**0.488**	0.040	0.168	0.240	0.164	0.000	**0.868**	0.088	**0.400**	0.200	0.168	0.400	0.028	0.372	0.540	0.172	0.052	**0.560**	0.288	0.132	0.388	**0.676**	0.172	0.112	0.024		0.000
**ROU**	0.000	0.000	0.000	0.000	0.000	0.000	0.000	0.000	0.000	0.000	0.000	0.000	0.000	0.000	0.000	0.000	0.000	0.000	0.000	0.000	**4.680**	0.000	0.000	0.000	0.000	0.000	0.000	0.000	0.000	0.000	0.000	

Note.– Cells in bold red indicate statistically significant pathways under the null hypothesis of random mixing population. Countries' codes are according to Figure 4. N. America: North America, C.S. America: Central & South America

B. Ratio of mean of observed over mean of expected migration events between large geographic regions and European countries

From \ To	N.America	C.S.America	Caribbean	Africa	Asia	Oceania	ALB	AUT	BEL	BGR	BLR	CHE	SRB	CYP	CZE/SVK	DEU	DNK	EST/LVA	ESP	FRA	GRC	ISR	ITA/IRL	LUX	NLD	FIN/NOR/SWE	POL	PRT	SVN	UKR	GBR	ROU
**N.America**		**3.317**	**1.829**	**2.362**	**1.538**	**2.059**	1.516	**1.474**	**1.226**	1.059	0.690	**3.281**	1.028	**2.142**	0.933	0.546	**1.887**	1.352	**1.374**	**2.876**	1.172	**1.546**	**2.113**	**2.192**	1.023	**1.310**	0.249	0.611	0.555	0.465	**3.633**	0.012
**C.S.America**	**3.113**		**1.992**	**2.210**	**1.532**	**1.987**	**2.619**	1.227	0.901	1.159	1.588	**2.969**	0.860	**2.197**	0.982	0.487	1.161	1.222	1.221	**2.506**	1.332	**1.855**	**1.991**	**2.124**	1.056	1.171	0.211	1.120	0.488	0.432	**2.962**	0.012
**Caribbean**	0.452	0.830		0.979	0.351	0.629	0.200	0.407	0.134	0.111	0.067	0.403	0.136	0.458	0.114	0.069	0.235	0.318	0.170	0.386	0.437	0.204	0.689	0.343	0.316	0.129	0.022	0.095	0.123	0.083	0.764	0.000
**Africa**	1.077	1.178	0.854		0.622	0.819	0.654	1.033	0.370	0.521	0.800	1.242	0.632	0.911	0.344	0.193	0.432	0.160	0.828	**1.719**	0.572	1.600	**1.652**	1.037	0.380	0.447	0.050	0.274	0.280	0.125	**1.679**	0.000
**Asia**	0.212	0.496	0.379	0.457		0.617	0.182	0.107	0.090	0.094	0.125	0.429	0.085	0.131	0.085	0.365	0.100	0.120	0.077	0.213	0.189	0.148	0.126	0.158	0.064	0.321	0.013	0.030	0.053	0.019	0.226	0.000
**Oceania**	0.678	0.846	0.854	0.419	1.086		0.333	0.487	0.295	0.171	0.444	0.741	0.209	0.746	0.500	0.383	0.451	0.333	0.303	0.644	0.361	0.273	0.809	0.476	0.592	0.381	0.172	0.104	0.174	0.058	1.170	0.006
**ALB**	0.167	0.250	0.000	3.000	0.500	0.000		0.000	0.200	1.000	-	5.500	1.000	0.500	0.500	1.000	0.000	0.000	0.000	0.500	4.667	-	5.500	0.000	2.000	0.000	0.000	-	1.500	-	1.000	0.000
**AUT**	0.396	0.391	0.254	0.729	0.123	0.452	0.571		0.637	0.203	0.000	0.857	0.722	0.373	0.992	0.448	0.570	0.053	0.398	0.726	0.082	0.481	0.759	1.371	1.386	0.795	0.275	0.623	0.378	0.088	0.979	0.000
**BEL**	0.258	0.263	0.113	0.156	0.077	0.256	0.136	0.921		0.090	0.815	0.599	0.456	0.281	0.776	**1.943**	1.056	0.062	**1.481**	0.216	0.054	0.423	0.978	**2.502**	**4.471**	0.936	0.451	**1.413**	0.487	0.054	0.385	0.000
**BGR**	0.107	0.157	0.077	0.130	0.101	0.043	0.000	0.098	0.211		5.000	0.240	0.140	0.179	0.063	0.093	0.172	0.200	0.086	0.213	0.047	0.182	0.092	0.089	0.048	0.141	0.444	0.078	0.043	0.467	0.273	0.087
**BLR**	-	-	-	0.000	1.000	0.000	-	-	0.000	-		-	-	0.000	0.000	-	-	-	-	-	-	-	-	-	-	-	0.000	-	-	-	-	-
**CHE**	**1.750**	**1.715**	0.975	**1.598**	0.654	1.139	**2.125**	1.091	1.332	0.755	**10.000**		1.485	1.068	0.547	0.534	1.091	1.692	1.239	**1.545**	0.971	1.538	**1.767**	1.248	1.388	0.686	0.174	0.554	0.506	0.215	**1.959**	0.000
**SRB**	0.217	0.355	0.264	0.524	0.164	0.132	0.000	0.754	0.280	**2.644**	0.000	0.888		0.400	0.438	**3.009**	0.363	2.000	0.448	0.406	0.304	0.467	0.312	0.284	0.265	0.408	0.060	0.531	**2.605**	0.056	0.435	0.000
**CYP**	0.688	0.643	0.427	1.375	0.323	0.558	1.111	0.466	0.341	0.756	1.000	1.119	0.297		**3.168**	0.311	0.414	0.545	**1.574**	0.768	**5.481**	0.286	0.391	0.925	0.488	0.413	0.091	0.233	0.167	0.179	1.087	0.000
**CZE/SVK**	0.101	0.131	0.051	0.064	0.034	0.102	0.065	0.443	0.229	0.080	0.063	0.110	0.093	0.123		0.371	0.161	0.065	0.270	0.123	0.046	0.187	0.215	0.152	0.310	0.220	0.096	0.189	0.226	0.015	0.091	0.000
**DEU**	0.195	0.204	0.115	0.101	0.059	0.315	0.110	**3.156**	**3.171**	0.814	0.115	0.593	**1.627**	0.990	**1.992**		**2.490**	**3.112**	**3.266**	0.606	0.072	1.408	**2.005**	1.011	**3.660**	**2.265**	**1.495**	**3.103**	1.018	0.112	0.777	0.004
**DNK**	0.351	0.408	0.240	0.296	0.150	0.297	0.145	0.622	**1.256**	0.141	0.067	0.495	0.412	1.122	0.474	0.692		0.091	0.744	0.443	0.145	0.500	0.789	0.382	1.112	**3.139**	0.278	0.606	0.259	0.038	0.520	0.016
**EST/LVA**	4.500	3.333	1.300	1.000	6.500	7.000	-	1.000	4.000	0.500	-	16.000	0.000	-	1.000	0.833	0.000		8.000	3.250	2.000	0.000	0.250	**85.000**	0.000	0.500	0.000	0.250	0.667	7.333	3.200	-
**ESP**	0.314	0.486	0.202	0.308	0.096	0.241	0.179	**1.536**	**2.608**	0.097	0.034	0.694	1.278	0.718	**1.559**	**2.835**	**1.858**	0.055		0.363	0.136	1.271	**2.063**	0.895	**3.069**	**2.633**	1.124	**3.231**	0.744	0.036	0.538	0.171
**FRA**	**2.456**	**2.455**	0.795	**1.765**	0.846	**1.312**	0.670	1.028	0.650	**1.433**	1.042	**1.792**	0.623	1.065	**1.344**	0.305	0.758	0.945	0.716		0.654	1.185	**1.271**	**1.316**	0.592	0.766	0.192	0.463	0.660	0.060	**1.815**	0.000
**GRC**	0.219	0.871	1.006	0.612	0.102	0.178	0.941	0.111	0.037	0.012	0.000	0.286	1.153	1.074	0.838	0.048	0.113	0.095	0.092	0.199		0.259	0.124	0.091	0.082	0.090	0.019	0.018	0.045	0.026	0.270	**14.420**
**ISR**	0.625	0.769	0.833	2.667	0.000	1.167	0.500	0.250	**26.500**	0.200	-	1.000	0.000	1.000	**17.000**	0.400	0.333	0.000	**13.000**	0.700	2.000		0.556	**26.667**	0.500	0.333	2.200	1.000	0.000	**-**	0.400	0.000
**ITA/IRL**	0.908	**1.360**	0.582	**2.852**	0.264	0.409	**1.919**	**3.938**	**1.320**	0.540	0.107	**2.275**	**1.488**	0.951	0.598	1.193	**1.621**	0.185	**1.255**	**1.951**	0.239	**1.547**		**1.555**	**1.686**	0.878	0.433	**1.371**	1.172	0.063	1.169	0.000
**LUX**	0.597	0.581	0.400	0.641	0.230	0.490	1.100	0.641	0.603	0.161	0.400	1.147	0.325	0.341	0.539	0.273	0.402	0.714	0.418	0.747	0.188	0.320	0.700		1.750	0.460	0.133	**2.773**	0.267	0.083	0.902	0.000
**NLD**	0.220	0.552	0.219	0.283	0.125	0.397	0.400	1.926	**7.720**	0.045	-	0.724	1.042	0.781	1.000	1.836	**2.433**	0.000	1.493	0.167	0.071	0.727	1.298	1.684		1.655	0.939	**2.579**	0.191	0.077	0.538	0.000
**FIN/NOR/SWE**	0.228	0.236	0.129	0.188	0.118	0.167	0.114	0.342	0.914	0.093	0.048	0.236	0.184	0.306	0.388	0.622	**1.264**	0.062	0.688	0.156	0.100	0.439	0.450	0.250	0.771		0.223	0.650	0.204	0.038	0.281	0.008
**POL**	0.021	0.035	0.014	0.010	0.007	0.018	0.000	0.402	0.223	0.921	0.476	0.067	0.063	0.032	0.467	0.899	0.124	**2.519**	0.567	0.038	0.770	1.210	0.111	0.289	0.214	0.719		0.210	0.032	0.962	0.032	0.000
**PRT**	0.281	0.260	0.097	0.167	0.086	0.176	0.167	**1.796**	**3.297**	0.130	0.333	0.634	1.198	0.552	1.082	**3.127**	**2.374**	0.000	**2.535**	0.281	0.119	1.742	**1.750**	**5.620**	**3.978**	**3.229**	0.962		0.574	0.000	0.358	0.000
**SVN**	0.084	0.068	0.047	0.079	0.036	0.077	0.000	**4.647**	0.161	0.191	0.000	0.132	**3.775**	0.157	0.767	0.644	0.161	0.000	0.089	0.094	0.000	0.075	0.097	0.105	0.087	**1.084**	0.034	0.072		0.000	0.156	0.000
**UKR**	0.522	1.600	**7.500**	0.357	0.095	0.143	0.333	0.167	**4.333**	**5.400**	**118.000**	1.875	0.083	**4.250**	**3.200**	2.150	0.550	**11.000**	2.000	0.333	**3.833**	**435.000**	0.316	**9.167**	0.000	**3.067**	**8.955**	0.154	0.000		0.231	0.000
**GBR**	**2.141**	**2.278**	1.465	**2.300**	0.894	**2.392**	1.111	1.024	0.923	1.323	0.000	**3.807**	0.595	**4.000**	0.714	0.824	1.587	1.000	1.409	1.849	1.387	1.625	**2.121**	1.946	1.500	1.406	**2.224**	0.843	0.667	0.316		0.000
**ROU**	0.000	0.000	0.000	0.000	0.000	0.000	0.000	0.000	0.000	0.000	0.000	0.000	0.000	0.000	0.000	0.000	0.000	0.000	0.000	0.000	**39.000**	0.000	0.000	0.000	0.000	0.000	0.000	0.000	0.000	0.000	0.000	

Note.– Cells in bold red indicate statistically significant pathways. The "–" corresponds to pathways with 0 mean of expected migration events and thus calculation of the ratio is not possible. Countries' codes are according to Figure 4. N. America: North America, C.S. America: Central & South America.
