# Supplementary material for: The global spread of HIV-1 subtype B epidemic
Source: Infect Genet Evol. 2016 Dec;46:169–79. doi: 10.1016/j.meegid.2016.05.041 (PMC5157885; doi:10.1016/j.meegid.2016.05.041)
Supplement: Supplemental Table 3 — A. Means of observed migration events across all bootstrap trees between the European countries. B. Ratio of mean of observed over mean of expected migration events between the European countries. [file mmc5.pdf]

Supplemental Table 3

A. Means of observed migration events across all bootstrap trees between the European countries

From \ To	ALB	AUT	BEL	BGR	BLR	CHE	SRB	CYP	CZE/SVK	DEU	DNK	EST/LVA	ESP	FRA	GRC	ISR	ITA/IRL	LUX	NLD	NOR/SWE/FIN	POL	PRT	SVN	UKR	GBR	ROU
ALB		0.000	0.000	0.020	0.000	0.044	0.000	0.000	0.004	0.000	0.000	0.000	0.000	0.008	0.044	0.000	0.048	0.004	0.000	0.000	0.000	0.000	0.000	0.000	0.008	0.000
AUT	0.004		**0.204**	0.004	0.000	**0.188**	0.104	0.048	0.248	0.264	0.196	0.000	0.156	**0.264**	0.000	0.028	**0.404**	**0.300**	**0.156**	0.252	0.064	**0.180**	0.148	0.000	0.012	0.000
BEL	0.000	**0.680**		0.016	0.020	**0.600**	**0.388**	0.180	**1.476**	**2.840**	**1.500**	0.000	**2.348**	0.304	0.012	0.092	**1.324**	**2.728**	**3.496**	**1.932**	**0.788**	**1.804**	0.388	0.012	0.100	0.000
BGR	0.000	0.012	0.004		0.008	0.008	0.016	0.004	0.008	0.008	0.000	0.000	0.008	0.028	0.004	0.000	0.008	0.004	0.000	0.000	**0.208**	0.000	0.000	0.008	0.008	0.000
BLR	0.000	0.000	0.000	0.000		0.000	0.000	0.000	0.000	0.000	0.000	0.000	0.000	0.000	0.000	**0.160**	0.000	0.000	0.000	0.000	0.000	0.000	0.000	0.000	0.000	0.000
CHE	0.056	0.092	**0.504**	0.052	0.060		0.168	0.060	0.060	0.052	0.128	0.000	**0.436**	0.420	0.064	0.044	**0.412**	0.060	0.160	0.064	0.004	0.080	0.036	0.004	0.096	0.000
SRB	0.000	**0.196**	0.048	**0.740**	0.000	0.056		0.032	0.156	**0.364**	0.068	**0.308**	0.100	0.048	0.024	0.016	0.100	0.020	0.044	0.092	0.024	0.068	**0.828**	0.000	0.024	0.000
CYP	0.004	0.012	0.052	0.016	0.000	0.068	0.012		**1.260**	0.004	0.040	0.012	**0.488**	0.128	**1.184**	0.004	0.036	0.040	0.032	0.036	0.024	0.008	0.000	0.004	0.048	0.000
CZE/SVK	0.008	**1.124**	1.056	0.032	0.004	0.032	0.424	0.260		**1.952**	0.728	0.000	1.392	0.156	0.056	0.184	**1.968**	0.248	**0.756**	0.980	0.632	0.712	**0.880**	0.068	0.068	0.000
DEU	0.004	**3.916**	**7.300**	**0.848**	0.000	0.324	**1.332**	**0.824**	**7.136**		**5.212**	**0.872**	**7.468**	0.336	0.060	**0.276**	**3.404**	**1.024**	**3.084**	**4.840**	**4.104**	**3.516**	**1.480**	0.008	0.200	0.020
DNK	0.004	**0.484**	**1.968**	0.040	0.000	0.132	0.284	**1.040**	**1.292**	**1.596**		0.008	**1.376**	0.240	0.048	0.136	**0.796**	0.152	**0.692**	**7.376**	0.432	**0.932**	0.224	0.000	0.048	0.016
EST/LVA	0.000	0.000	0.000	0.000	0.000	0.016	0.000	0.004	**0.064**	0.000	0.000		0.008	0.036	0.004	0.000	0.020	**0.072**	0.004	0.000	0.000	0.000	0.000	0.036	0.000	0.000
ESP	0.044	**4.320**	**11.908**	0.060	0.004	**0.816**	**2.864**	**1.040**	**8.424**	**14.928**	**9.280**	0.016		0.572	0.256	**0.856**	**11.820**	**1.824**	**5.860**	**12.880**	**6.728**	**10.828**	**2.244**	0.000	**0.544**	0.168
FRA	0.068	0.424	0.288	**0.440**	0.008	**0.752**	0.188	0.364	**1.200**	0.228	0.396	0.012	0.880		0.324	0.084	0.816	**0.524**	0.104	0.588	0.052	0.260	0.216	0.012	**0.512**	0.004
GRC	0.016	0.000	0.016	0.004	0.000	0.056	**0.328**	0.104	**0.404**	0.004	0.000	0.000	0.016	0.032		0.000	0.028	0.008	0.004	0.036	0.012	0.008	0.008	0.012	0.012	**2.608**
ISR	0.004	0.008	**0.408**	0.000	0.008	0.008	0.004	0.004	**0.360**	0.004	0.004	0.000	**0.624**	0.000	0.004		**0.384**	**0.216**	0.008	0.008	**0.088**	0.004	0.004	**0.108**	0.004	0.000
ITA/IRL	**0.420**	**3.828**	**2.316**	0.200	0.000	**1.976**	**0.660**	0.240	**1.240**	**2.044**	**2.392**	0.020	**2.288**	**3.028**	0.096	**0.284**		**1.388**	**1.428**	**1.924**	**0.928**	**1.572**	**1.120**	0.008	**0.372**	0.000
LUX	0.008	0.108	0.108	0.004	0.004	**0.324**	0.052	0.004	0.076	0.224	0.040	0.004	0.136	0.212	0.012	0.016	0.116		**0.236**	0.120	0.012	**1.016**	0.024	0.000	0.024	0.000
NLD	0.004	**0.128**	**1.576**	0.000	0.004	0.032	**0.100**	0.056	**0.396**	**0.432**	**0.548**	0.000	**0.416**	0.012	0.004	0.060	**0.332**	0.048		**0.384**	**0.132**	**0.432**	0.096	0.000	0.000	0.000
NOR/SWE/FIN	0.016	**0.620**	**2.708**	0.024	0.000	0.236	0.376	0.140	1.412	**2.588**	**4.304**	0.000	**2.388**	0.132	0.048	0.192	**1.500**	0.260	**0.836**		0.640	**1.504**	0.348	0.004	0.300	0.028
POL	0.000	**0.464**	0.440	**1.196**	0.032	0.076	0.056	0.036	**1.932**	**2.016**	0.336	**0.664**	**1.428**	0.020	**1.208**	**0.740**	0.560	**0.696**	0.184	**1.968**		0.236	0.092	**0.864**	0.016	0.000
PRT	0.004	**0.484**	**2.084**	0.012	0.000	0.112	**0.328**	0.072	**0.864**	**2.156**	**1.228**	0.000	**2.232**	0.120	0.020	0.080	**0.852**	**2.032**	**1.160**	**2.160**	**0.608**		**0.312**	0.000	0.020	0.000
SVN	0.000	**1.980**	0.096	0.032	0.000	0.056	**1.864**	0.004	**1.640**	**1.068**	0.060	0.000	0.064	0.028	0.008	0.000	0.172	0.036	0.044	**1.016**	0.024	0.040		0.000	0.012	0.000
UKR	0.000	0.004	**0.172**	0.048	**1.324**	0.012	0.004	0.064	**0.348**	0.000	0.000	0.016	0.088	0.008	**0.200**	**1.240**	**1.124**	**0.164**	0.000	0.052	**0.848**	0.000	0.000		0.016	0.000
GBR	0.000	0.008	0.028	0.020	0.000	**0.624**	0.004	0.072	0.036	0.044	0.044	0.000	0.072	0.096	0.032	0.000	0.076	0.048	0.020	0.040	**0.772**	0.020	0.012	0.004		0.000
ROU	0.000	0.000	0.000	0.000	0.000	0.000	0.004	0.000	0.000	0.000	0.000	0.000	0.000	0.000	**4.560**	0.000	0.000	0.000	0.000	0.000	0.000	0.000	0.000	0.000	0.000	

Note.– Cells in bold red indicate statistically significant pathways under the null hypothesis of random mixing population. Countries' codes are according to Figure 4.

B. Ratio of mean of observed over mean of expected migration events between the European countries

From \ To	ALB	AUT	BEL	BGR	BLR	CHE	SRB	CYP	CZE/SVK	DEU	DNK	EST/LVA	ESP	FRA	GRC	ISR	ITA/IRL	LUX	NLD	NOR/SWE/FIN	POL	PRT	SVN	UKR	GBR	ROU
ALB		-	-	-	-	11.000	-	-	1.000	0.000	0.000	-	-	0.667	-	0.000	-	1.000	0.000	0.000	-	-	0.000	-	-	0.000
AUT	1.000		**5.667**	0.091	0.000	**3.917**	2.364	1.714	2.583	2.200	2.579	0.000	1.560	**3.300**	0.000	1.167	**3.607**	**10.714**	**7.800**	2.423	0.696	**5.625**	2.643	0.000	0.429	0.000
BEL	0.000	**3.269**		0.114	-	**2.542**	**1.865**	1.098	**2.906**	**9.467**	**4.261**	0.000	**5.060**	0.800	0.079	1.917	**3.343**	**16.634**	**24.278**	**4.200**	**2.402**	**8.352**	1.672	0.176	0.714	0.000
BGR	0.000	0.600	0.063		-	0.286	0.500	0.143	0.125	0.111	0.000	0.000	0.118	0.636	0.200	0.000	0.154	0.250	0.000	0.000	**4.333**	0.000	0.000	0.500	0.286	0.000
BLR	-	-	-	-		-	-	-	-	-	-	-	-	-	-	**-**	-	-	-	-	-	-	-	-	-	-
CHE	4.667	0.920	**3.073**	0.867	15.000		2.333	0.682	0.197	0.260	0.615	0.000	**2.137**	1.842	0.889	1.222	**2.641**	0.625	2.500	0.302	0.024	0.571	0.281	0.167	1.143	0.000
SRB	0.000	**5.444**	0.632	**26.429**	-	0.875		0.571	1.625	**3.500**	0.739	**19.250**	0.962	0.414	0.316	0.800	1.087	0.333	1.222	0.697	0.300	1.700	**11.500**	0.000	0.600	0.000
CYP	0.500	0.375	0.867	0.333	-	1.063	0.273		**14.318**	0.063	0.435	0.750	**3.697**	1.391	**32.889**	0.200	0.643	0.714	1.000	0.375	0.231	0.143	0.000	0.250	1.333	0.000
CZE/SVK	0.118	**2.322**	1.347	0.088	0.200	0.056	1.000	0.644		**2.291**	0.831	0.000	1.172	0.156	0.120	1.278	**2.299**	0.646	**2.392**	0.911	0.702	1.309	**1.732**	0.415	0.202	0.000
DEU	0.077	**21.756**	**17.892**	**4.818**	0.000	1.038	**7.400**	**3.433**	**10.372**		**13.862**	**27.250**	**11.180**	0.706	0.242	**3.632**	**7.400**	**4.339**	**16.404**	**8.521**	**9.243**	**14.898**	**5.286**	0.091	1.220	0.179
DNK	0.143	**2.814**	**5.928**	0.333	0.000	0.550	1.775	**7.879**	**2.543**	**5.051**		0.200	**3.044**	0.588	0.316	2.429	**2.187**	0.974	**4.943**	**16.613**	1.113	**4.396**	0.836	0.000	0.414	0.160
EST/LVA	-	-	-	-	-	-	0.000	-	**-**	-	0.000		-	-	-	0.000	5.000	**-**	-	-	-	-	0.000	-	-	0.000
ESP	0.524	**9.391**	**15.345**	0.176	0.143	**1.672**	**6.450**	**2.921**	**7.043**	**16.513**	**11.100**	0.267		0.656	0.566	**5.350**	**12.468**	**4.750**	**17.035**	**11.541**	**7.157**	**18.291**	**4.250**	0.000	**1.813**	0.609
FRA	2.125	1.656	0.550	**2.444**	0.500	**2.265**	0.644	1.338	**1.531**	0.380	0.739	0.200	1.134		1.157	1.000	1.417	**1.819**	0.765	0.907	0.094	0.942	0.740	0.150	**2.667**	0.030
GRC	1.000	0.000	0.138	0.077	-	0.609	**6.308**	2.600	**2.730**	0.033	0.000	0.000	0.091	0.250		0.000	0.259	0.080	0.100	0.237	0.107	0.111	0.080	0.500	0.300	**65.200**
ISR	-	2.000	**51.000**	-	-	2.000	1.000	-	**30.000**	0.500	-	-	**156.000**	0.000	1.000		**48.000**	**-**	1.000	1.000	**22.000**	-	-	**-**	-	-
ITA/IRL	**8.750**	**16.220**	**5.908**	-	0.000	**6.676**	**3.235**	1.395	**2.366**	**4.563**	**5.806**	0.313	**4.366**	**6.105**	0.381	**3.944**		**7.229**	**11.516**	**3.272**	**2.128**	**5.311**	**3.836**	0.065	**2.163**	0.000
LUX	0.400	2.700	1.929	0.100	1.000	**3.522**	1.444	0.143	0.655	2.154	0.400	0.250	1.133	1.432	0.188	2.000	1.813		**8.429**	1.000	0.158	**23.091**	0.316	0.000	0.545	0.000
NLD	1.000	**32.000**	**39.400**	0.000	-	2.000	**25.000**	2.000	**12.375**	**5.684**	**9.786**	0.000	**8.667**	0.375	0.091	3.750	**8.300**	3.000		**6.857**	**5.500**	**13.500**	4.000	0.000	0.000	0.000
NOR/SWE/FIN	0.286	**1.914**	**3.782**	0.087	0.000	0.454	1.011	0.365	1.342	**3.851**	**6.184**	0.000	**2.477**	0.169	0.108	1.171	**1.761**	0.631	**3.800**		0.816	**3.418**	0.744	0.036	0.938	0.132
POL	0.000	**2.035**	1.122	**6.102**	2.000	0.288	0.326	0.188	**3.019**	**4.667**	0.800	**18.444**	**2.231**	0.042	**4.794**	**18.500**	1.429	**2.949**	1.211	**3.302**		1.054	0.371	**10.800**	0.105	0.000
PRT	0.333	**5.261**	**28.944**	0.300	-	1.120	**3.905**	1.000	**3.323**	**18.586**	**8.297**	0.000	**14.308**	0.789	0.217	3.333	**7.607**	**29.882**	**20.714**	**11.489**	**3.535**		**3.900**	0.000	0.294	0.000
SVN	0.000	**26.053**	0.727	0.348	0.000	0.609	**22.190**	0.040	**6.406**	**7.629**	0.349	0.000	0.291	0.175	0.111	0.000	1.075	0.450	0.647	**5.184**	0.102	0.313		0.000	0.188	0.000
UKR	-	0.500	**10.750**	-	**-**	1.000	0.500	16.000	**17.400**	0.000	0.000	-	3.143	1.000	**50.000**	**-**	**93.667**	**41.000**	0.000	4.333	**70.667**	0.000	-		-	-
GBR	0.000	0.333	0.875	2.500	-	**31.200**	0.200	3.000	0.643	0.786	1.100	0.000	1.286	1.600	2.667	0.000	1.188	1.714	1.000	0.435	**17.545**	1.250	0.429	0.167		0.000
ROU	-	0.000	0.000	0.000	-	0.000	0.500	0.000	0.000	0.000	0.000	0.000	0.000	0.000	**190.000**	-	0.000	0.000	0.000	0.000	0.000	0.000	0.000	0.000	0.000	

Note.– Cells in bold red indicate statistically significant pathways. The "–" corresponds to pathways with 0 mean of expected migration events and thus calculation of the ratio is not possible. Countries' codes are according to Figure 4.
