# Supplementary material for: The global spread of HIV-1 subtype B epidemic
Source: Infect Genet Evol. 2016 Dec;46:169–79. doi: 10.1016/j.meegid.2016.05.041 (PMC5157885; doi:10.1016/j.meegid.2016.05.041)
Supplement: Supplemental Table 4 — List of studies fulfilling selection criteria. [file mmc6.pdf]

Supplemental Table 4. List of studies fulfilling selection criteria.

Continent	Country	Country Code	Pubmed ID	1st author
America	United States of America	USA	9780274	Young B
		USA	11153669	Parkin NT
		USA	11574914	Gonzales MJ
		USA	11741169	Machado DM
		USA	12131189	Simon V
		USA	12167680	Little SJ
		USA	15181563	Weinstock HS
		USA	16760795	Henry K
		USA	17083034	Viani RM
		USA	17502727	Eshleman SH
		USA	18353964	Little SJ
		USA	19036810	Wang YE
		USA	19398922	Ellis GM
	Canada	CAN	20622676	Ragonnet CM
	Mexico	MEX	20624071	Vázquez VE
	Argentina, Brazil, Bahamas, Mexico	ARG, BRA, BHS, MEX	17147509	Gomez CM
	Cuba	CUB	16910827	Perez L
	Cuba	CUB	12172086	Cuevas MT
	Jamaica	JAM	20929350	Roye ME
	Dominican Republic, Jamaica, Haiti, Trinidad & Tobago	DOM, JAM, HTI, TTO	19279683	Nadai Y
	Belize, Bolivia, Ecuador, El Salvador, Honduras, Panama, Paraguay, Peru, Puerto Rico, Uruguay	BLZ, BOL, ECU, SLV, HND, PAN, PRY, PER, PRI, URY	22132104	Junqueira DM
	Trinidad & Tobago, Antigua & Barbuda, Dominica, Grenada, Saint Lucia, Guyana, Saint Vincent and Grenadines, Suriname, Montserrat	TTO, ATG, DMA, GRD, LCA, GUY, VCT, SUR, MSR	14601595	Vaughan HE
	Colombia	COL	16607004	Sanchez GI
	Venezuela	VEN	19327054	Rangel HR
	Venezuela	VEN	16562642	Dieudonne M
	Chile	CHL	17457921	Rios M
	Argentina	ARG	17961105	Dilernia DA
	Argentina	ARG	16773027	Petroni A
	Brazil	BRA	19689200	de Sa-Filho DJ
	Brazil	BRA	19682948	Cardoso LP
	Brazil	BRA	18327988	de Sa-Filho DJ
	Brazil	BRA	15725753	Sa Filho DJ
	Brazil	BRA	21417758	Carvalho BC
	Brazil	BRA	20087934	Cardoso LP
Africa	Senegal	SEN	19553825	Ndiaye HD
	Sudan	SDN	12402957	Hierholzer M
	Morocco	MAR	21087198	Annaz HE
	Algeria	DZA	16623641	Bouzeghoub S
	South Africa	ZAF	18593350	Jacobs GB
	Seychelles	SYC	17604537	Razafindratsimandresy R
	Madagascar	MDG	16796535	Razafindratsimandresy R
Asia	Thailand	THA	21449850	Praparattanapan J
	Taivan	TWN	21235402	Kao CF
	South Korea	PRK	21062223	Cho YK
	Japan	JPN	17194486	Gatanaga H
	Iran	IRN	20610954	Hamkar R
	Hong-Kong	HKG	20063993	Tsui SK
	China	CHN	18834324	Wang W
	China	CHN	19453981	Tu YQ
	China	CHN	17678466	Zhong P
	China	CHN	21830915	Ye JR
Oceania	Australia	AUS	21806486	Chibo D

Note.− Continent and Country refer to the origin of the population study. Country codes are according to ISO.
